# ATGL-mediated lipid droplet lipolysis promotes collective cell migration in *Drosophila*

**DOI:** 10.1242/dev.205486

**Published:** 2026-08-20

**Authors:** Israel J. Wipf, Emma J. Lowden, Michelle S. Giedt, Julia R. Dorale, Kennedy F. Godfredsen, Tina L. Tootle

**Affiliations:** Department of Biology, University of Iowa, Iowa City, IA 52242, USA

**Keywords:** Migration, Lipid droplets, Lipolysis, *Drosophila*, Border cells, Delamination

## Abstract

Although lipid droplets (LDs), organelles central to lipid and energy homeostasis, are implicated in cancer cell migration, their roles during collective cell migration remain unknown. We use *Drosophila* border cell (BC) migration as an *in vivo* model of invasive, collective migration to dissect the roles of LDs and the conserved LD lipase Adipose Triglyceride Lipase (ATGL). LDs undergo dynamic changes and exhibit a polarized distribution during BC migration. Loss of ATGL increases LD volume, whereas BC ATGL overexpression depletes LDs. Loss, knockdown or overexpression of ATGL delay migration and impair delamination. Additionally, loss of ATGL disrupts BC mitochondria functions, including membrane potential. Similarly, loss of the mitochondrial fatty acid importer carnitine palmitoyltransferase 1 (CPT1) reduces mitochondrial membrane potential and severely blocks delamination. These results demonstrate that tight regulation of lipid mobilization from LDs, including its role in energy production, drives delamination and collective migration. These findings not only suggest how cancer cells may exploit LDs to promote their invasive behaviors but also highlight the crucial role of LDs in migration during development, hinting at their broader significance in diverse migratory contexts.

## Teaser

**Summary:** Using *Drosophila* border cells as a model, this study provides *in vivo* evidence that lipid droplets and an associated lipase promote delamination and invasive collective migration.

## INTRODUCTION

Collective cell migration is essential for embryonic development and wound healing but is co-opted during cancer metastasis (Carmona-Fontaine et al., 2008; Cheung et al., 2013; Riahi et al., 2015; Padmanaban et al., 2019). Evidence supports that metastases are often seeded by collectively migrating cancer cells, as the presence of circulating tumor cell clusters is associated with worse prognosis (Murlidhar et al., 2017; Wang et al., 2017a). It is therefore crucial to understand the mechanisms governing collective migration to facilitate wound healing and combat cancer.

Collective migration is an energy-intensive process. An important issue is how cells produce sufficient adenosine triphosphate (ATP) to fuel migration (Zanotelli et al., 2018; Mosier et al., 2021). While proliferative cancer cells prefer glycolysis for ATP production (Warburg et al., 1927), evidence supports that metastasizing cancer cells depend on mitochondrial lipid oxidation to fuel migration (LeBleu et al., 2014; Cunniff et al., 2016; Lin et al., 2019). Consequently, the synthesis, storage and utilization of lipids must be properly coordinated for efficient cell movement.

Lipid storage and utilization are regulated by lipid droplets (LDs), dynamic organelles comprising a phospholipid monolayer surrounding a core of neutral lipids (e.g. triglycerides and sterol esters; Walther and Farese, 2012; Welte, 2015; Olzmann and Carvalho, 2019). LDs are formed at the endoplasmic reticulum (ER) (Kalantari et al., 2010; Jacquier et al., 2011) and are safe reservoirs for fatty acids (FAs), which could otherwise cause membrane damage and cellular dysfunction (Lee et al., 1994; Nguyen et al., 2017). LDs supply organelles with lipids for diverse metabolic needs. FAs can be rapidly mobilized from LDs by lipolysis: the hydrolysis of triglyceride into glycerol and free FAs (Walther and Farese, 2012; Welte, 2015; Olzmann and Carvalho, 2019). LD lipolysis is mediated by a cascade of lipases, including adipose triglyceride lipase (ATGL), hormone-sensitive lipase (HSL) and monoglyceride lipase (Zimmermann et al., 2004). Mobilized FAs serve as building blocks for membrane lipids and signaling molecules or oxidative substrates for β-oxidation and ATP production (Walther and Farese, 2012; Welte, 2015; Olzmann and Carvalho, 2019).

We use the migration of border cells (BCs) during *Drosophila* oogenesis as an *in vivo* model of invasive, collective migration. Each *Drosophila* ovary consists of 15-20 chains of developing follicles called ovarioles. There are 14 stages of follicle development. Within each follicle there are 15 nurse cells, which support the developing oocyte, surrounded by ∼650 follicle cells (Giedt and Tootle, 2023). During Stage 9 (S9), 6-8 follicle cells are specified as BCs, detach from the anterior follicular epithelium and collectively migrate between the nurse cells to the oocyte*.*

In this study, we sought to determine the role of LDs during BC migration. LDs are present within the BC cluster and undergo changes in both number and volume as migration proceeds. We observe smaller LDs in late migration, suggesting LD lipids are being mobilized during migration. Further, the LDs exhibit a polarity within the cluster; there are fewer LDs in the front. These findings led us to explore the role of the lipase ATGL (*Drosophila* Brummer, Bmm) in BC migration. Our data support the model that ATGL promotes lipid mobilization from LDs; ATGL loss increases LD volume within the BC cluster, whereas BC overexpression of ATGL depletes LDs within the cluster. Importantly, genetic loss, BC knockdown or BC overexpression of ATGL leads to migration defects – delayed migration and impaired delamination. These findings suggest that the BCs are sensitive to the amount of ATGL present, and that delamination and collective migration depend on proper storage and utilization of LD-stored lipids. Our data support the model that these lipids (FAs) are trafficked to the mitochondria for β-oxidation; loss of ATGL alters mitochondrial morphology and distribution, decreases membrane potential relative to organelle mass, and accumulates ROS in the mitochondria in the BC cluster. Further, FA import into the mitochondria is essential for BC migration, as loss of the importer carnitine palmitoyltransferase 1 (CPT1; *Drosophila* Withered, Whd) prevents delamination and impairs mitochondrial membrane potential. This work is the first to demonstrate that tight regulation of mobilization and utilization of stored lipids from LDs is essential for successful *in vivo* delamination and migration. Given the ubiquity of LDs as lipid storage organelles, precise regulation over LD storage, mobilization and utilization is likely a conserved requirement in diverse migratory contexts across species.

## RESULTS

### LDs are dynamic within the BC cluster during migration

**Fig. 1.**
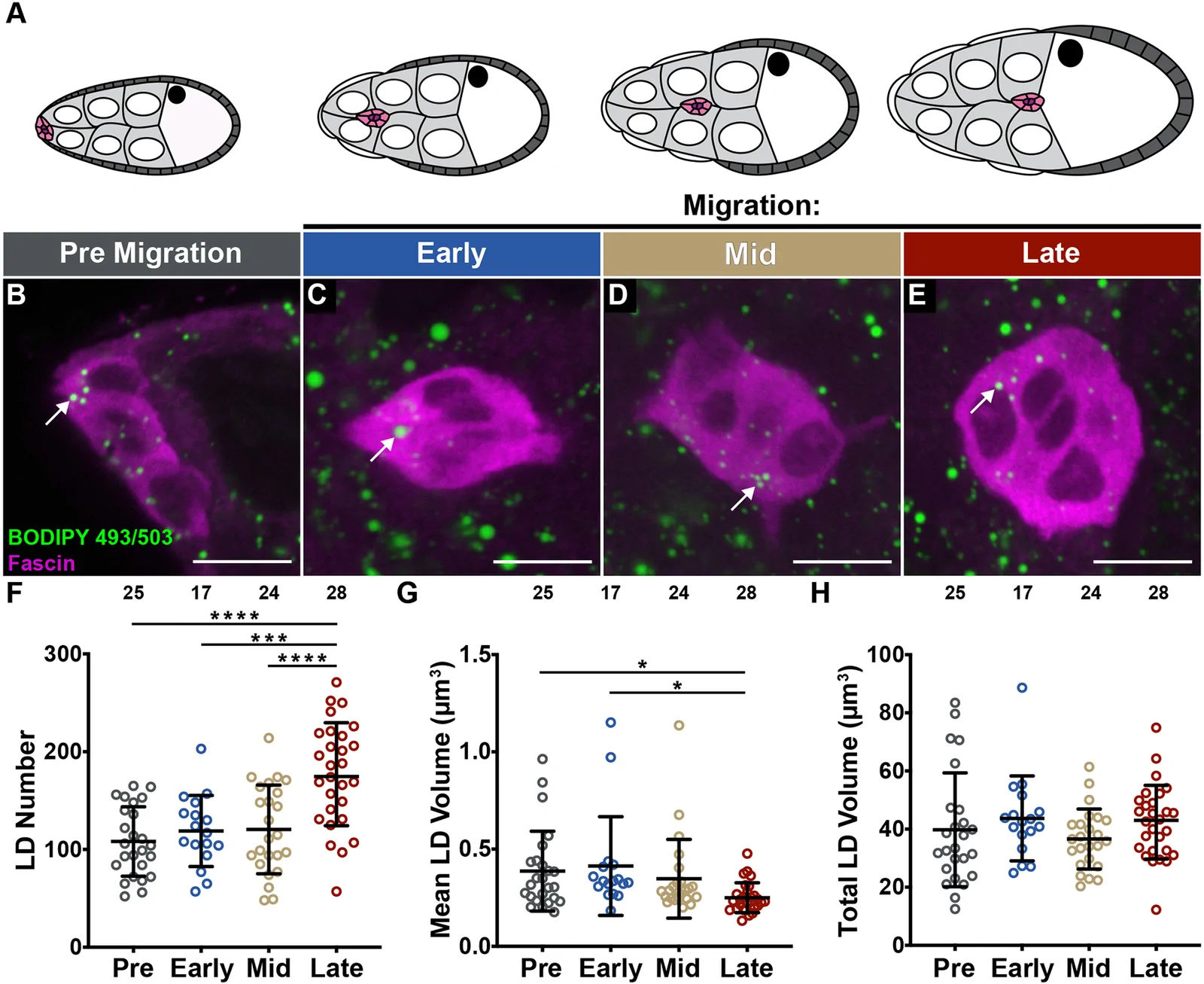
**Border cell lipid droplets are dynamic throughout migration.** (A) Schematic of border cell (BC) migration. The BCs (pink), polar cells (purple), outer follicle cells (dark gray), stretch follicle cells (white), nurse cells (light gray with white nuclei) and oocyte (white with black nucleus) are shown. (B-E) Maximum projections of three confocal slices of wild-type (*yw*) BCs at the indicated stages of migration (A) stained for lipid droplets (LDs) (BODIPY 493/503; green) and Fascin (BCs; magenta). White arrows indicate examples of LDs in the BC clusters. Images are brightened by 30%. Black boxes have been placed behind panel labels. Scale bars: 10 μm. (F-H) Quantification of LD number (F), mean volume (G) and total volume (H) in the BC clusters at the indicated stages of migration. *n,* number of clusters (listed at the top of each set of data); line indicates the mean; error bars indicate s.d. ns, *P*>0.05; *P<0.05, ***P<0.001, ****P<0.0001 (one-way ANOVA with Tukey multiple comparison test). During S9, the BCs delaminate from the follicular epithelium and collectively migrate between the nurse cells to the nurse cell-oocyte boundary; at the same time the follicle grows (A). LDs are present within the BC cluster prior to and throughout migration (B-E). LD number increases (F) and mean LD volume decreases (G) within the BC cluster during late migration relative to earlier stages of migration, while total LD volume in the cluster remains unchanged (H).

To determine whether LDs are present in the BCs, we labeled both the BC cluster and LDs. We detect LDs within the BC cluster throughout migration (Fig. 1A-E). Pre-migratory BCs appear to contain fewer, large LDs, while BCs later in migration contain numerous small LDs (Fig. 1B-E). To assess these differences, we quantified the number and size of LDs within the BC cluster before and throughout migration. The number of LDs (∼115) remains consistent within the BC cluster until late migration, whereupon LD number (∼177) increases compared to pre-, early and mid-migration (Fig. 1F; *P*<0.0001). This result could indicate that, later in migration, LD synthesis is increased or LD utilization is decreased, or both. Supporting that stored lipids are released from LDs throughout migration, LD volume decreases during late migration (Fig. 1G; ∼0.25 μm^3^) relative to pre- (∼0.39 μm^3^; *P*<0.05) and early migration (∼0.41 μm^3^; *P*<0.05). However, there is no detectable change in total lipid volume in the BCs across migration (Fig. 1H; ∼40 μm^3^); this result is consistent with BC LDs increasing in number yet decreasing in size throughout migration. To rule out the possibility that these changes in LD number and volume are due to changes in the cluster size, we measured the size of the BC cluster at each point in migration and found there are no differences (Fig. S1). Finally, we labeled the polar cells and LDs, and find the polar cells contain a few LDs of average size (Fig. S2; ∼0.40 μm^3^; ∼4/cell), revealing that most LDs within the cluster are in the BCs. Together, these data reveal that LDs are not only present within the BCs before and throughout migration but undergo dynamic changes in number and size.

### LDs exhibit spatial polarity within the BC cluster

**Fig. 2.**
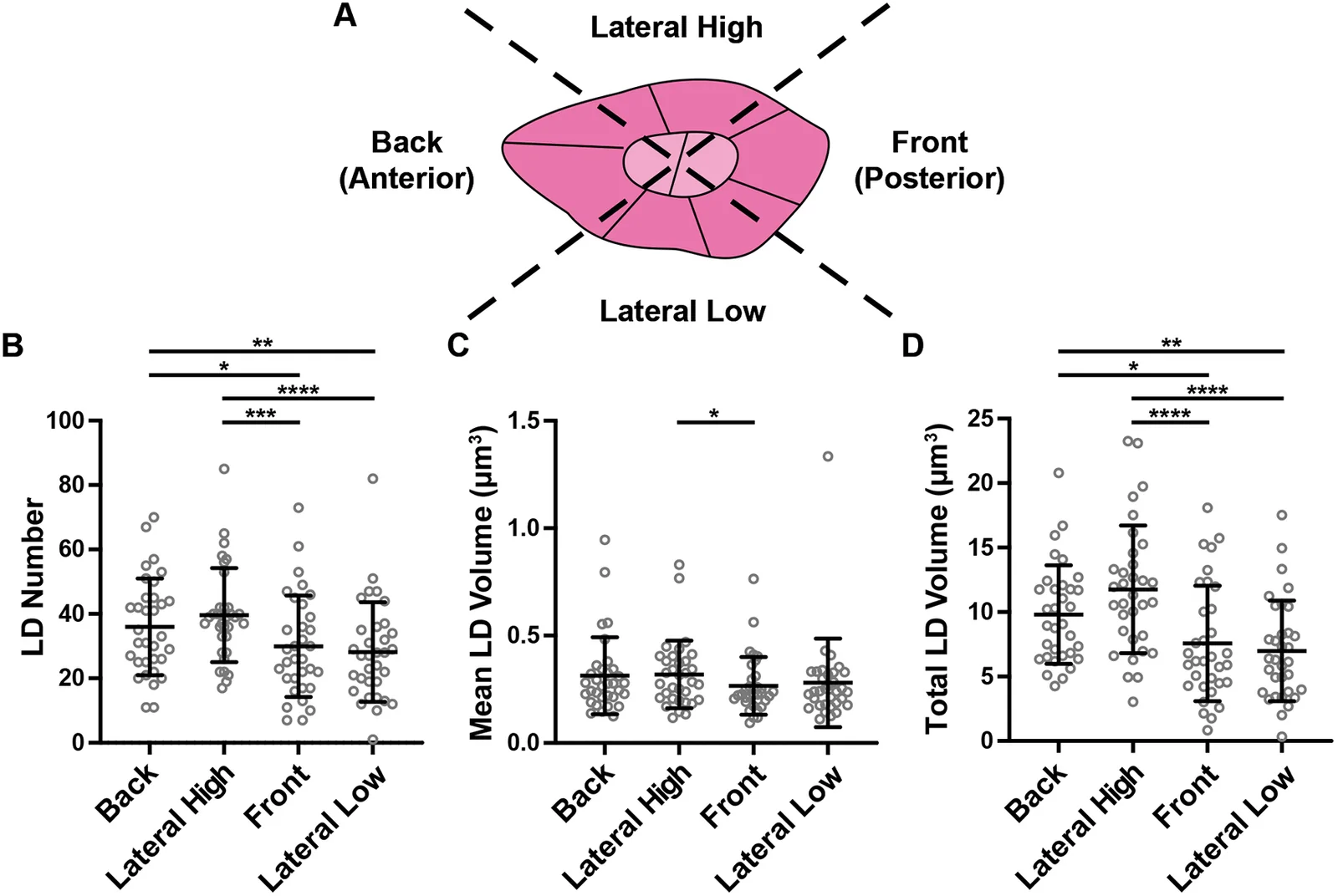
**Lipid droplet distribution is polarized within the border cell cluster.** (A) Schematic demonstrating the spatial analysis of lipid droplets (LDs) within four regions of the border cell (BC) cluster (pink): back (anterior), lateral high, front (posterior) and lateral low. (B-D) Quantification of LD number (B), mean volume (C) and total volume (D) within the four regions of wild-type (*yw*) BC clusters from the images used in Fig. 1. *n=*33 clusters; line indicates the mean; error bars indicate s.d. ns, *P*>0.05; *P<0.05, **P<0.01, ***P<0.001, ****P<0.0001 (one-way ANOVA with Tukey multiple comparison test). LDs show spatial polarization within the BC cluster, with reduced LD number and total volume in the front and lateral low regions (B,D). Mean LD volume is reduced in the front compared to the lateral high region (C).

To determine if the LDs are uniformly distributed or exhibit a polarity within the BC cluster, we divided the cluster into four regions – back, two lateral sides and front – and quantified LD parameters. LDs exhibit a polarity within the cluster (Fig. 2). The two lateral regions within a cluster had one region with more LDs than the other, and, therefore, we specified each cluster as having one lateral high and one lateral low region (Fig. 2A). The front (*P*<0.05 and *P*<0.001) and lateral low regions (*P*<0.01 and *P*<0.001) within the BC clusters exhibit fewer LDs (Fig. 2B) and decreased total LD volume (Fig. 2D; front, *P*<0.05 and *P*<0.0001; lateral low, *P*<0.01 and *P*<0.0001) compared to the back and lateral high regions, respectively. In contrast, mean LD volume is decreased only in the front versus the lateral high region (Fig. 2C, *P*<0.01). We speculate the LD polarity reflects spatial differences in LD usage (high in front and lateral low) and formation (high in back and lateral high), and that leader cells are replaced by cells with more LDs (lateral high) following lipid depletion.

### ATGL regulates LD volume in the BC cluster

**Fig. 3.**
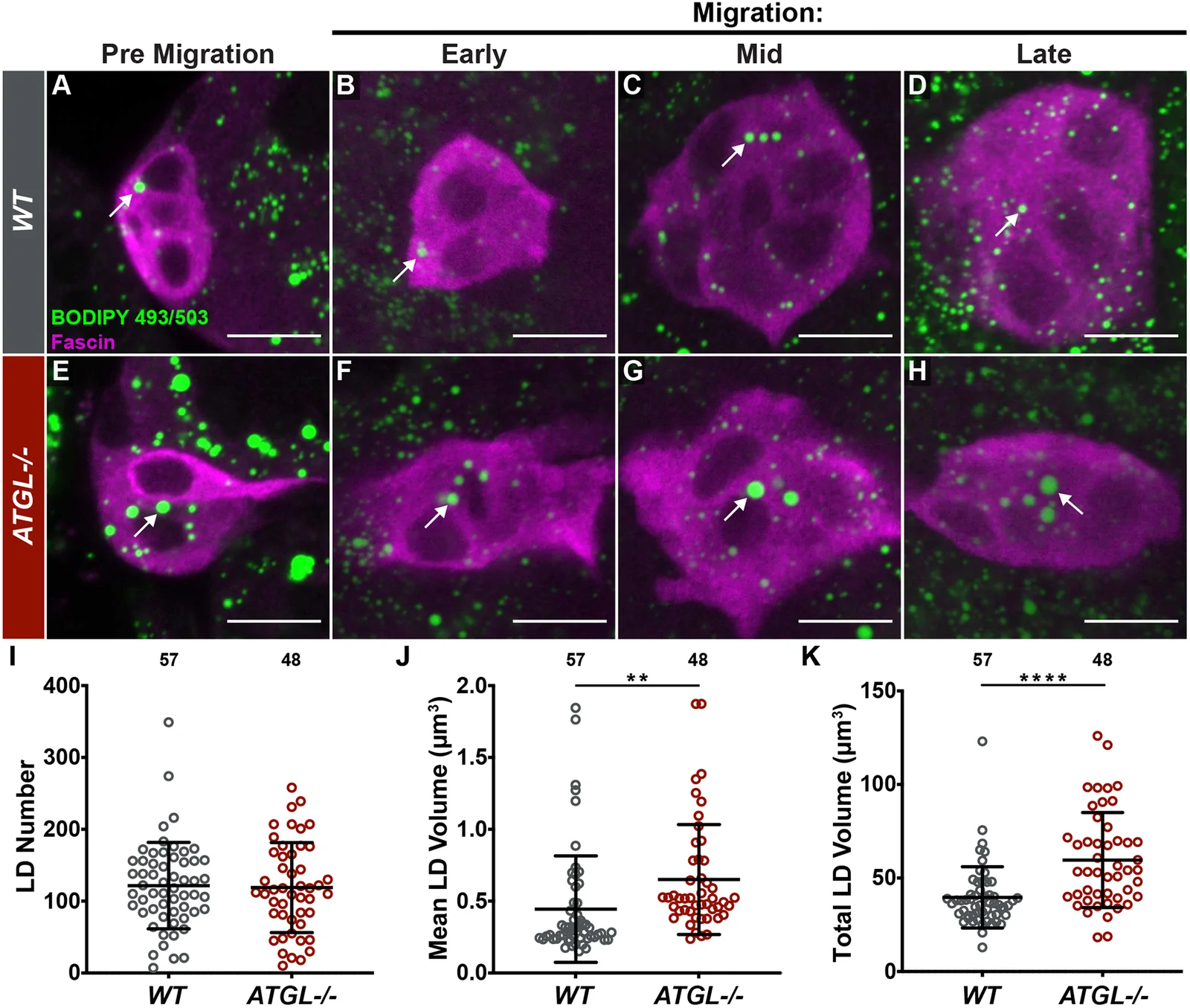
**ATGL regulates lipid droplet volume in the border cell cluster.** (A-H) Maximum projections of three confocal slices of S9 follicles with the border cells (BCs) at the indicated stage of migration stained for lipid droplets (LDs) (BODIPY 493/503; green) and Fascin (BCs; magenta). White arrows indicate examples of LDs in the BC cluster. Images are brightened by 30% (B and H are brightened by 50%). Black boxes have been placed behind panel labels. Scale bars: 10 μm. (A-D) Wild type (*yw*). (E-H) *ATGL^−/−^* (*bmm^1^/bmm^1^*). (I-K) Quantification of LD number (I), mean volume (J) and total volume (K) in the BC clusters for the indicated genotypes. *n*, number of clusters (listed at the top of each set of data); line indicates the mean; error bars indicate s.d. ns, *P*>0.05; **P<0.01, ****P<0.0001 (unpaired *t*-test, two-tailed). Loss of ATGL results in larger LDs within the BC cluster relative to wild type throughout migration (A-H). Loss of ATGL does not affect LD number (I) but increases both mean volume (J) and total volume (K) within the BC cluster.

We next investigated the role of ATGL, a key lipolysis enzyme (Gronke et al., 2005). Loss of ATGL (null) results in visibly larger LDs within the BC cluster throughout migration (Fig. 3A-H; Movie 1). This result suggests that lipolysis and lipid utilization from LDs within the BCs are decreased when ATGL is lost. Quantification of the LDs reveals that while the number of LDs within the BC cluster remains unchanged (Fig. 3I), loss of ATGL increases mean LD volume (Fig. 3J; *P*<0.01) and total lipid volume (Fig. 3K; *P*<0.0001). These results may be due, in part, to an increased BC cluster volume in *ATGL* mutants (Fig. S3A, *P*<0.05). Analyzing BC morphology reveals this change in volume is not due to changes in length or width of the cluster but is due to increased height (Fig. S3B-D; *P*<0.05). Alternatively, excess lipid storage may expand BC cluster volume. These data suggest ATGL is a crucial regulator of LD lipolysis in the BC cluster.

### ATGL promotes BC detachment and on-time migration

**Fig. 4.**
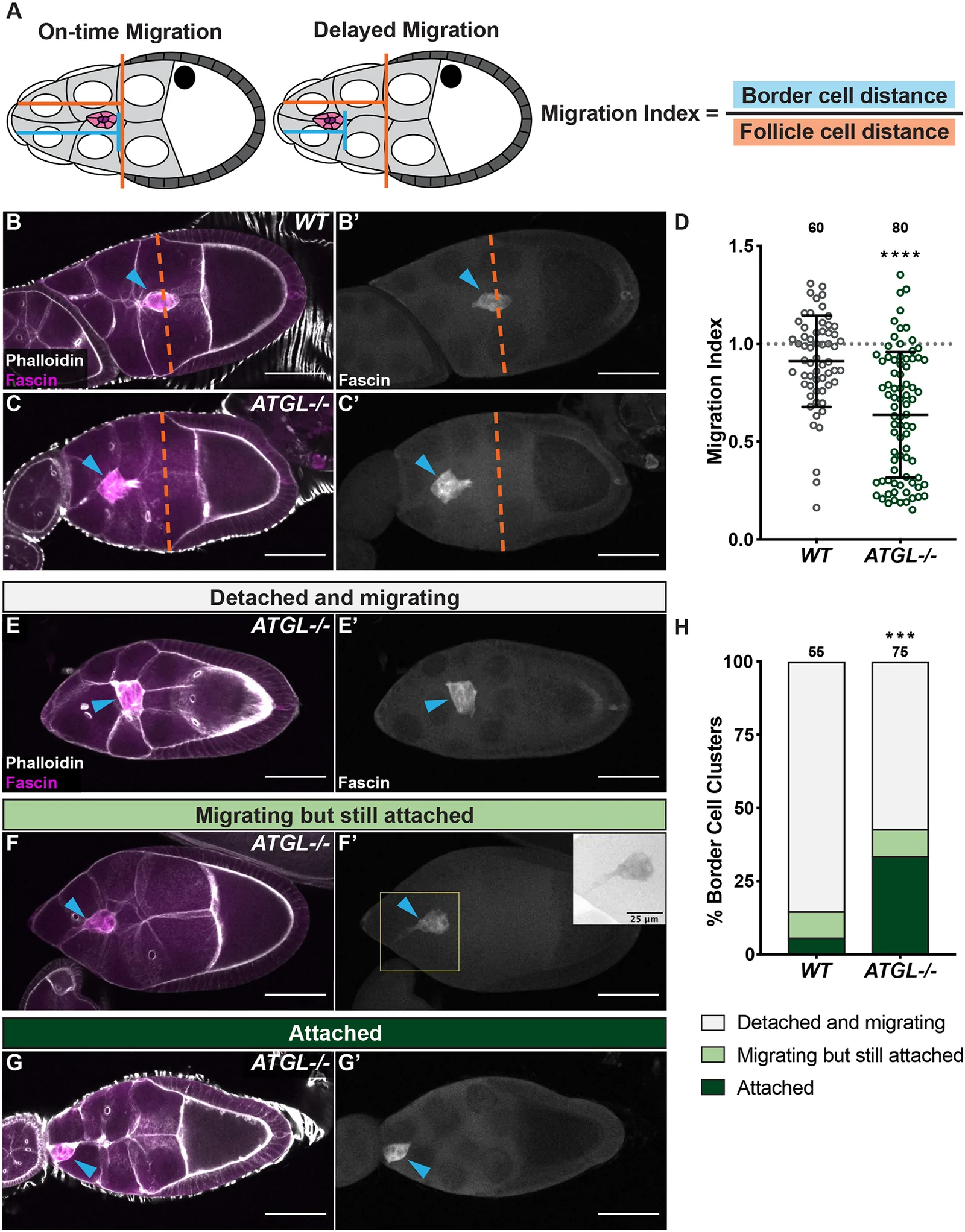
**ATGL promotes on-time border cell migration and detachment.** (A) Schematic of on-time (left) and delayed (right) border cell (BC) migration, and the formula for calculating the migration index. The BCs (pink), polar cells (purple), outer follicle cells (dark gray), stretch follicle cells (white), nurse cells (light gray with white nuclei) and oocyte (white with black nucleus) are shown. The migration index is calculated by dividing the distance of BCs (blue line) by the distance of the outer follicle cells from the anterior end of the follicle (orange line). (B-C′) Maximum projections of three confocal slices of S9 follicles stained for Fascin (BCs; magenta) and F-actin (phalloidin; white). Blue arrowheads indicate the BC cluster. Orange dashed lines indicate the position of the outer follicle cells. Images are brightened by 30%. Black boxes have been placed behind panel and channel labels. Scale bars: 50 μm. (B,B′) Wild type (*yw*). (C,C′) *ATGL^−/−^* (*bmm^1^/bmm^1^*). (D) Graph of the migration index for the indicated genotypes. *n*, number of follicles (listed at the top of each set of data); dotted line indicates on-time migration; solid line indicates the mean; error bars indicate s.d., ns, *P*>0.05; ****P<0.0001 (unpaired *t*-test, two-tailed). (E-G′) Maximum projections of three confocal slices of *ATGL^−/−^* S9 follicles showing BC clusters that are detached from the follicular epithelium and migrating (E,E′), migrating but still attached (F,F′) and attached (G,G′). Blue arrowheads indicate the BC cluster. (H) Quantification of BC detachment defects for the indicated genotypes. BC clusters categorized as detached and migrating (light gray), migrating but still attached (light green) or attached (dark green). *n,* number of clusters (listed at the top of each set of data); ***P<0.001 (Pearson's Chi-squared test). The timing of BC migration can be assessed during S9 by comparing the distance the BCs have migrated relative to the position of the outer follicle cells (A). In wild-type follicles, the BC cluster detaches from the follicular epithelium and migrates; during migration, the cluster remains in-line with the outer follicle cells (A, left; B,B',D,H). Loss of ATGL delays BC migration during S9 (C-D) and impairs detachment of the cluster from the follicular epithelium (E-H).

We next asked whether ATGL regulates BC invasion and migration. To assess BC migration during S9, we compare the position of the BC cluster relative to the position of the outer follicle cells (Fig. 4A). In wild-type follicles, the cluster remains in-line with the outer follicle cells throughout migration (Fig. 4B,B′). Loss of ATGL delays BC migration, as the cluster is anterior to the outer follicle cells (Fig. 4C,C′). To quantify BC migration, we measured the distance the BC cluster had migrated from the anterior end of the follicle (BC distance) and divided it by the distance of the outer follicle cells from the end of the follicle (follicle cell distance); this calculation is termed the migration index (Fig. 4A; Fox et al., 2020; Lamb et al., 2020; Mellentine et al., 2023). A migration index of ∼1 indicates on-time migration, while values less than 1 indicate delayed migration. The migration index in *ATGL* mutants is below 1 (MI=0.637; *P*<0.0001) and lower than controls (Fig. 4D; MI=0.911). These data indicate that ATGL promotes on-time BC migration.

As many *ATGL* mutant follicles exhibited extremely low migration index values, we next investigated the role of ATGL in BC detachment or delamination. In contrast to wild-type follicles, in *ATGL* mutants the BC clusters often fail to detach from the follicular epithelium. To quantify defects in BC detachment, we classified mid- to late-S9 follicles into three categories: those with BC clusters that are fully detached and migrating (Fig. 4E,E′), those migrating but maintaining a tail attached to the epithelium (Fig. 4F,F′) and those failing to migrate and remaining fully attached to the epithelium (Fig. 4G,G′). Loss of ATGL resulted in 33.3% of BC clusters remaining attached to the follicular epithelium, compared to 5.5% in controls (Fig. 4H; *P*<0.001). Only 57.3% of *ATGL* mutant clusters were detached and migrating, compared to 85.5% in controls (Fig. 4H; *P*<0.001). These data indicate that ATGL contributes to BC delamination, suggesting that the *ATGL* mutant S9 migration delay is due, at least partially, to a failure to detach from the follicular epithelium.

We next sought to determine whether other LD-associated proteins also facilitate BC migration. We assessed the roles of five additional LD-associated proteins: Perilipin 1 (PLIN1), PLIN2, HSL, comparative gene identification 58 (CGI-58) and Jabba. Surprisingly, none of these proteins are required for on-time BC migration (Fig. S4). These results indicate the role of ATGL during BC migration is likely specific.

### ATGL RNAi knockdown in the BCs exhibits minor changes in LDs

Given the increased LD volume within the BC clusters of *ATGL* mutants, we asked whether we could detect changes in LDs when we used the UAS/GAL4 system to knockdown ATGL by RNAi in the BCs. RNAi knockdown of ATGL in the BCs does not alter BC LD volume (Fig. S5A-C,E,F). However, knockdown of ATGL increases LD number (Fig. S5D) compared to the GAL4-only control (*P*=0.053) but not compared to the RNAi-only control. The difference in BC LD phenotypes between *ATGL* mutants and BC-specific ATGL RNAi could be due to insufficient knockdown or indicate that ATGL also functions in other cells.

### ATGL is required in the BCs to promote detachment and migration

We next sought to determine if ATGL is required in the BCs to promote detachment and on-time migration. RNAi knockdown of ATGL in the BCs delays their migration (Fig. S6A-D; MI=0.575) compared to the GAL4-(MI=0.929; *P*<0.0001) and RNAi-only (MI=0.834; *P*<0.001) controls. Furthermore, RNAi knockdown of ATGL in the BCs results in significant detachment defects. BC knockdown of ATGL resulted in 39% of BC clusters remaining attached to the follicular epithelium (Fig. S6E), compared to 12.5% and 10% in the GAL4- (*P*<0.05) and RNAi-only (*P*<0.01) controls, respectively. Together, these data reveal that ATGL is required in the BCs for promoting delamination and on-time migration.

### ATGL overexpression in the BCs depletes LDs

**Fig. 5.**
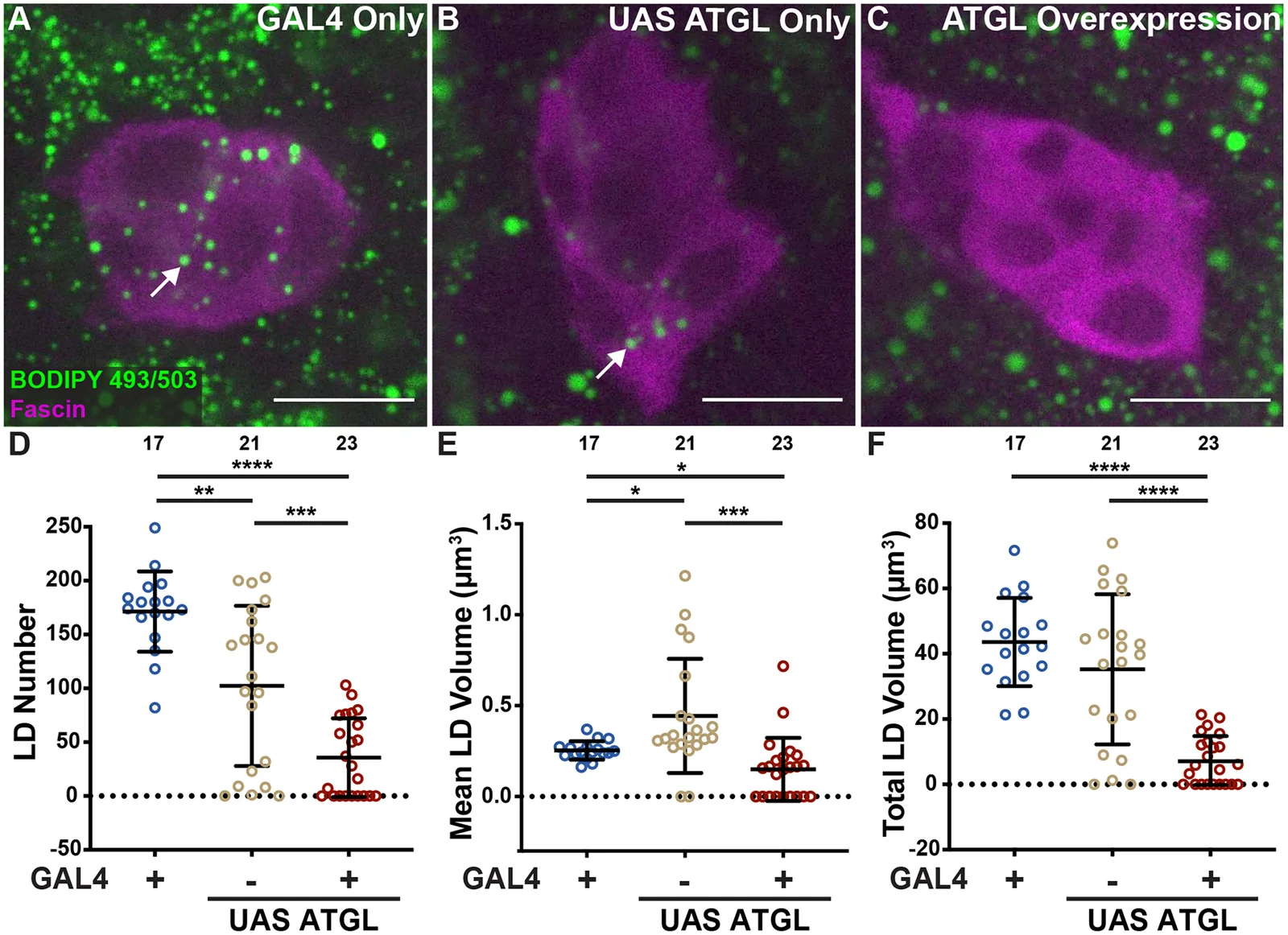
**Overexpression of ATGL in the border cells depletes lipid droplets.** (A-C) Maximum projections of three confocal slices of S9 follicles stained for lipid droplets (LDs) (BODIPY 493/503; green) and Fascin [border cells (BCs); magenta]. White arrows indicate examples of LDs in the BC cluster. Images are brightened by 60%. Black boxes have been placed behind channel labels. Scale bars: 10 μm. (A) GAL4 control (*slbo GAL4/+*). (B) UAS ATGL control (*UAS bmm/+*). (C) BC overexpression of ATGL (*slbo GAL4/UAS bmm*). (D-F) Quantification of LD number (D), mean volume (E) and total volume (F) in the BC clusters for the indicated genotypes. *n*, number of clusters (listed at the top of each set of data); line indicates the mean; error bars indicate s.d.; ns, *P*>0.05; *P<0.05, **P<0.01, ***P<0.001, ****P<0.0001 (one-way ANOVA with Tukey multiple comparison test). Overexpression of ATGL in the BCs decreases LD number (A-D), moderately decreases mean volume (E) and depletes total volume (F) in the BCs.

Overexpression of the lipase HSL depletes LDs and decreases invasive potential in pancreatic cancer cells (Rozeveld et al., 2020). We asked whether the BCs might be similarly sensitive to the amount of ATGL present. To test this, we used the UAS/GAL4 system to overexpress ATGL in the BCs. While BC LDs of GAL4-only controls appear normal (Fig. 5A), UAS ATGL-only control BCs appear moderately depleted of LDs (Fig. 5B). Strikingly, however, BC overexpression of ATGL strongly depletes BC LDs (Fig. 5C). Quantification reveals, as the images suggest, fewer LDs in UAS-only BC clusters compared to the GAL4-only control (∼102 compared to ∼171, respectively; *P*<0.001). One explanation for this result is that the UAS ATGL expresses the transgene in the absence of GAL4. To test this possibility, we analyzed BC cluster LDs in either a wild-type or *ATGL* heterozygous background combined with UAS ATGL (no GAL4). We reasoned that if the reduction of LDs observed in UAS ATGL control clusters is due to increased ATGL protein, then genetically reducing ATGL may rescue the phenotype. Indeed, genetic reduction of ATGL restores the number of BC cluster LDs back to normal levels (Fig. S7). Regardless of the leaky UAS ATGL expression in the absence of GAL4, BC overexpression of ATGL dramatically decreases LD number in the BCs (Fig. 5D; ∼36) compared to both the GAL4- (∼171; *P*<0.0001) and UAS-only (∼102; *P*<0.001) controls. Further, BC overexpression of ATGL moderately decreases mean LD volume (Fig. 5E; ∼0.16 μm^3^) compared to the GAL4- (∼0.25 μm^3^; *P*<0.05) and UAS-only (∼0.43 μm^3^; *P*<0.001) controls, and dramatically decreases total lipid volume (Fig. 5F; ∼7.29 μm^3^) compared to the GAL4- (∼43.59 μm^3^; *P*<0.0001) and UAS-only (∼35.23 μm^3^; *P*<0.0001) controls. This near loss of BC cluster LDs when ATGL is overexpressed could be due to decreased LD biogenesis or to increased mobilization of LD lipids, or both. Given the lipase function of ATGL, it is likely that ATGL overexpression is excessively releasing lipids from LDs, leading to LD shrinkage and eventual depletion. Together, these data support that ATGL levels and activity must be tightly regulated in the BCs to control LD number and size.

### ATGL overexpression in the BCs delays detachment and migration

**Fig. 6.**
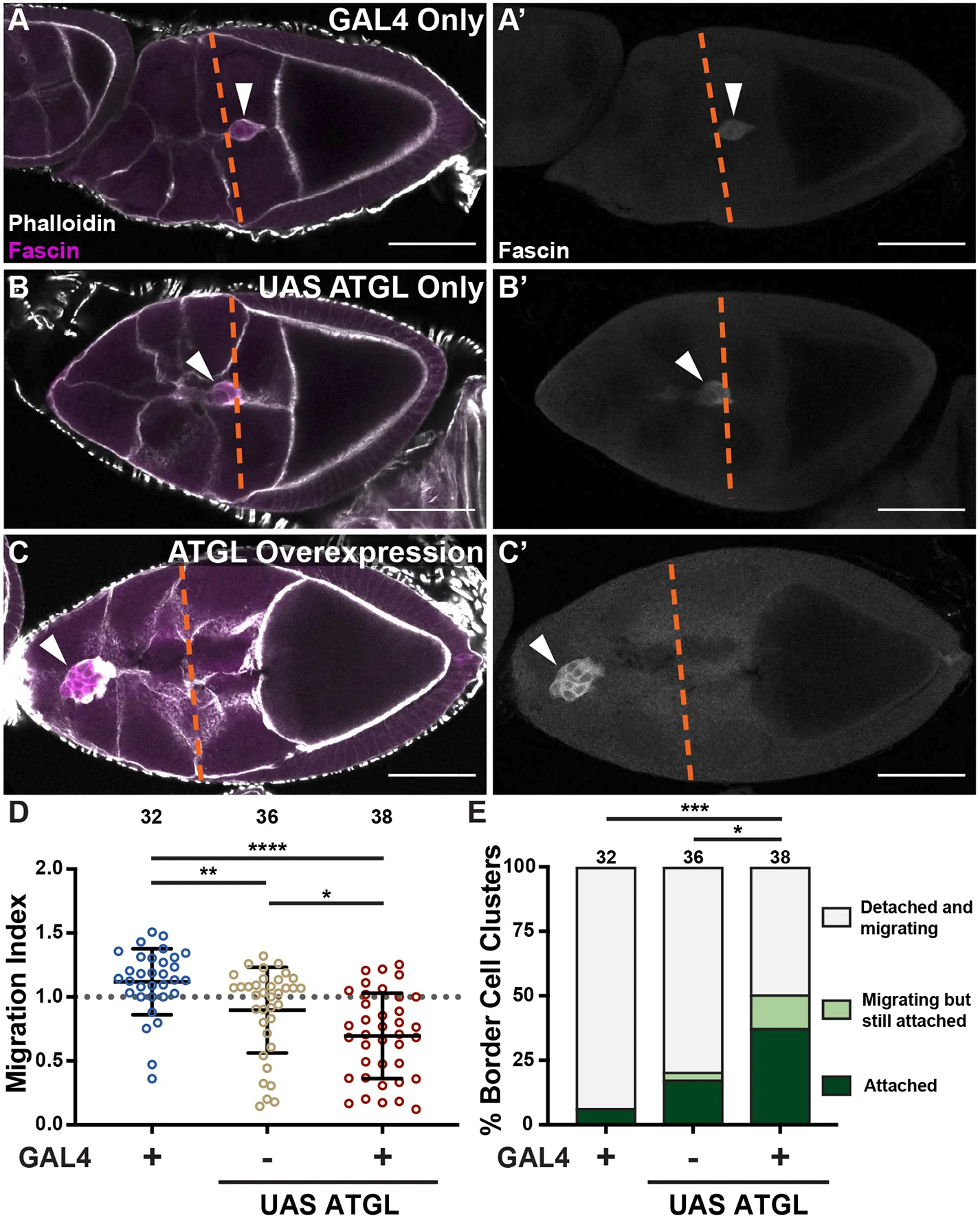
**Overexpression of ATGL in the border cells impairs detachment and delays migration.** (A-C′) Maximum projections of three confocal slices of S9 follicles stained for Fascin [border cells (BCs); magenta] and F-actin (phalloidin; white). White arrowheads indicate the BC cluster. Orange dashed lines indicate the position of the outer follicle cells. Images are brightened by 50%. Black boxes have been placed behind panel labels. Scale bars: 50 μm. (A,A′) GAL4 control (*slbo GAL4/+*). (B,B′) UAS ATGL control (*UAS bmm/+*). (C,C′) BC overexpression of ATGL (*slbo GAL4/UAS bmm*). (D) Graph of the migration index for the indicated genotypes. *n,* number of follicles (listed at the top of each set of data); dotted line indicates on-time BC migration; solid line indicates the mean; error bars indicate s.d.; *P<0.05, **P<0.01, ****P<0.0001 (one-way ANOVA with Tukey multiple comparison test). (E) Quantification of BC detachment defects for the indicated genotypes. BC clusters categorized as detached and migrating (light gray), migrating but still attached (light green) or attached (dark green). *n*, number of clusters (listed at the top of each set of data); ns, *P*>0.05; *P<0.05, ***P<0.001 (Pearson's Chi-squared test with Monte Carlo simulation). BC overexpression of ATGL delays BC migration (A-D) and impairs detachment of the cluster from the follicular epithelium (E).

We next assessed how ATGL overexpression in the BCs affects delamination and migration. ATGL overexpression in the BCs delays their migration (Fig. 6A-D; MI=0.696) compared to the GAL4- (MI=1.118; *P*<0.0001) and UAS-only (MI=0.917; *P*<0.05) controls. Additionally, BC ATGL overexpression results in 37% of BC clusters remaining attached to the follicular epithelium (Fig. 6E), compared to 6% and 17% in the GAL4- (*P*<0.001) and UAS-only (*P*<0.05) controls, respectively. Because the severity of the detachment and migration defects correlates with the extent of LD depletion observed within each genotype (Fig. 5), these findings support the model that proper lipid storage and utilization are essential for both on-time delamination and migration, and loss of lipid stores within the BC cluster impairs these processes.

### ATGL regulates mitochondrial morphology and function in the BC cluster

How do ATGL-mobilized lipids promote BC delamination and on-time migration? There are many possible mechanisms, as mobilized lipids could serve as precursors for membrane lipids and/or signaling molecules, or as substrates for energy production.

First, we tested the possibility that ATGL-mediated lipolysis is releasing the FA arachidonic acid for prostaglandin (PG) production, as it does during S10B of *Drosophila* oogenesis (Giedt et al., 2023). PG signaling is required for on-time BC migration (Fox et al., 2020; Mellentine et al., 2023). To test whether the control of ATGL over BC migration is dependent on PG signaling, we performed a dominant genetic interaction assay between ATGL and dCOX1 (Pxt), the *Drosophila* cyclooxygenase-like enzyme responsible for all PG synthesis (Tootle and Spradling, 2008). We can perform this assay because heterozygosity for the individual mutations (*ATGL^−/+^* or *dCOX1^−/+^*) does not appreciably affect BC migration (Fig. S8). If ATGL acts upstream of PG synthesis, then the double heterozygotes should exhibit delayed migration. The BCs from double heterozygotes of *ATGL* and *dCOX1* migrate on time (Fig. S8), suggesting that FAs mobilized by ATGL are acting through a mechanism other than PG production to promote BC migration.

Given that migration is an energy-intensive process, we next sought to address the possibility that mobilized FAs are provided to the mitochondria for β-oxidation and energy production. Compared to wild-type BC mitochondria, which appear evenly dispersed throughout the cytoplasm, mitochondria in *ATGL* mutants cluster in large aggregates (Fig. S9A-B′). Additionally, we labeled the ER and found no obvious differences in ER morphology in the BC clusters between wild type and *ATGL* mutants (Fig. S9C-D′), suggesting that loss of ATGL may not result in global lipid dysregulation in the BCs but rather causes a mitochondrial specific effect.

**Fig. 7.**
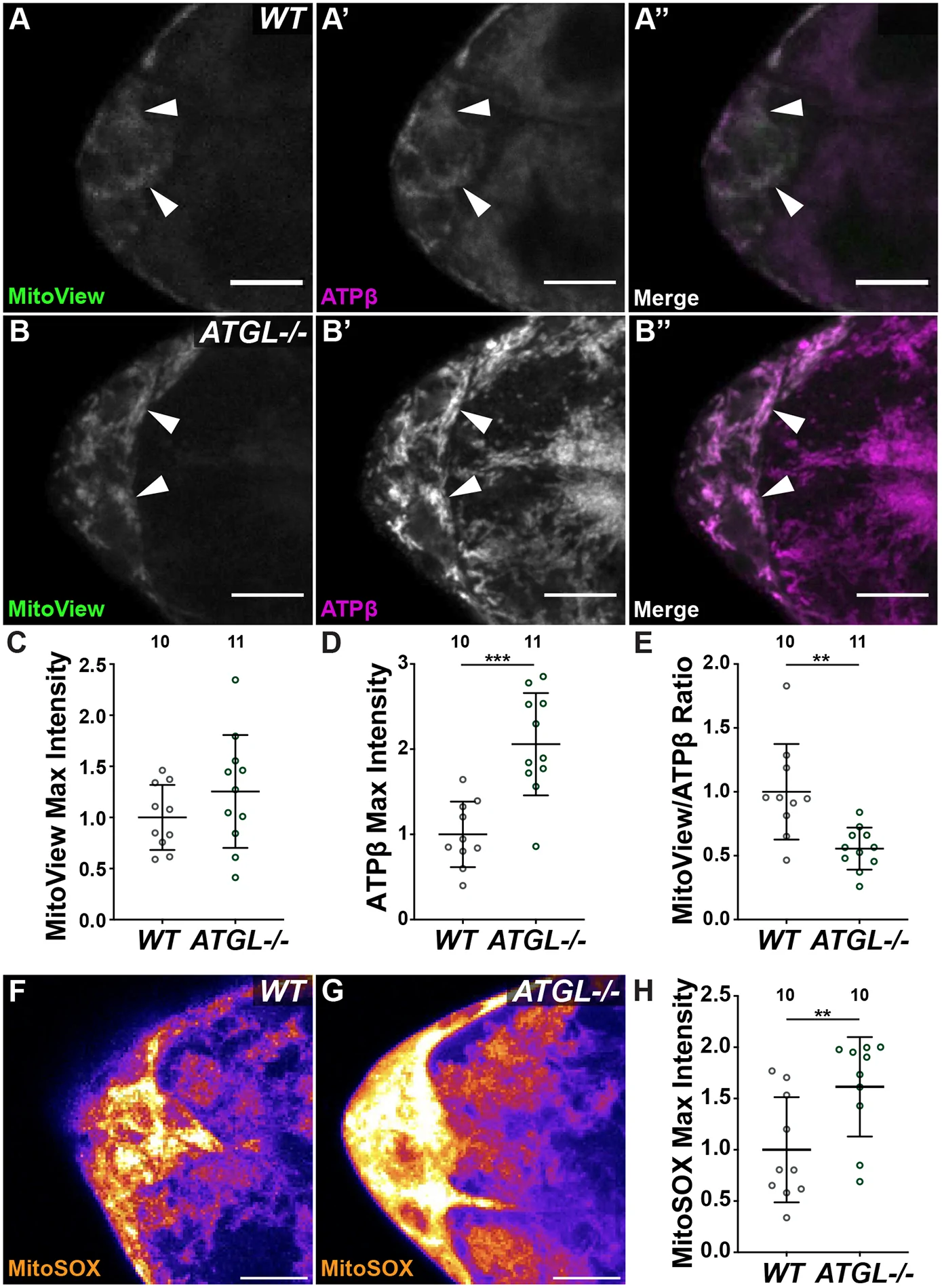
**ATGL promotes mitochondrial function in the border cells.** (A-B″) Maximum projections of three confocal slices of S9 follicles stained for total mitochondria (ATPβ; magenta in merge) and oxidatively active mitochondria (MitoView; green in merge). White arrowheads indicate instances of colocalization between ATPβ and MitoView in the border cell (BC) cluster. Images have been brightened by 50%. Black boxes have been placed behind panel labels. Scale bars: 10 μm. (A-A″) Wild type (*yw*). (B-B″) *ATGL^−/−^* (*bmm^1^/bmm^1^*). (C-E) Quantification of maximum fluorescence intensity of MitoView (C), maximum fluorescence intensity of ATPβ (D) and ratio of MitoView to ATPβ (E) in the BC cluster for the indicated genotypes. *n*, number of clusters (listed at the top of each set of data); line indicates the mean; error bars indicate s.d.; ns, *P*>0.05; **P<0.01, ***P<0.001 (unpaired *t*-test, two-tailed). (F,G) Maximum projections of three confocal slices of S9 follicles stained for MitoSOX (Fire LUT to indicate intensity). Black boxes have been placed behind panel labels. Scale bars: 10 μm. (F) Wild type (*yw*). (G) *ATGL^−/−^* (*bmm^1^/bmm^1^*). (H) Quantification of maximum fluorescence intensity of MitoSOX signal in the BC cluster for the indicated genotypes. *n*, number of clusters (listed at the top of each set of data); line indicates the mean; error bars indicate s.d. **P<0.01 (unpaired *t*-test, two-tailed). Loss of ATGL disrupts mitochondrial morphology and function in the BCs (A-D). Loss of ATGL results in reduced membrane potential relative to total mitochondria (E) and increased ROS in the BCs (F-H).

As mitochondrial clustering is indicative of mitochondrial dysfunction, we asked whether loss of ATGL impairs mitochondrial function. An indicator of mitochondrial health is the maintenance of inner membrane potential, which is essential for ATP production. To assess mitochondrial membrane potential, we employed MitoView, a mitochondrial stain whose localization depends on mitochondrial polarization. MitoView localizes to mitochondria in the BCs (Fig. 7A-A″), and loss of ATGL does not significantly change the intensity of the staining (Fig. 7B-C). To use this stain to assess mitochondrial function, we normalized mitochondrial membrane potential to total mitochondrial mass by calculating the ratio of polarization-dependent (MitoView) to polarization-independent mitochondrial signal (ATP-β). Loss of ATGL increases the maximum intensity of ATP-β staining (Fig. 7D; *P*<0.001) and decreases the ratio of polarization-dependent to polarization-independent staining (Fig. 7E; *P*<0.01). This result suggests that BCs in *ATGL* mutants have reduced overall mitochondrial membrane potential, consistent with reduced energy production.

Given that mitochondrial mass fails to provide a proportional increase in total membrane potential in *ATGL* mutants, we investigated whether there is oxidative stress. MitoSOX was used to detect mitochondrial superoxide, a byproduct of respiration that is toxic at high concentrations and a primary member of the reactive oxygen species (ROS) family. Loss of ATGL increases MitoSOX fluorescence in the BC cluster (Fig. 7F-H; *P*<0.01). Together, these data support a role for ATGL in maintaining BC mitochondrial function and health, as its loss alters mitochondrial morphology, impairs membrane potential and increases oxidative stress. These defects could be due to impaired FA β-oxidation.

### CPT1 is required for mitochondrial function and BC detachment

**Fig. 8.**
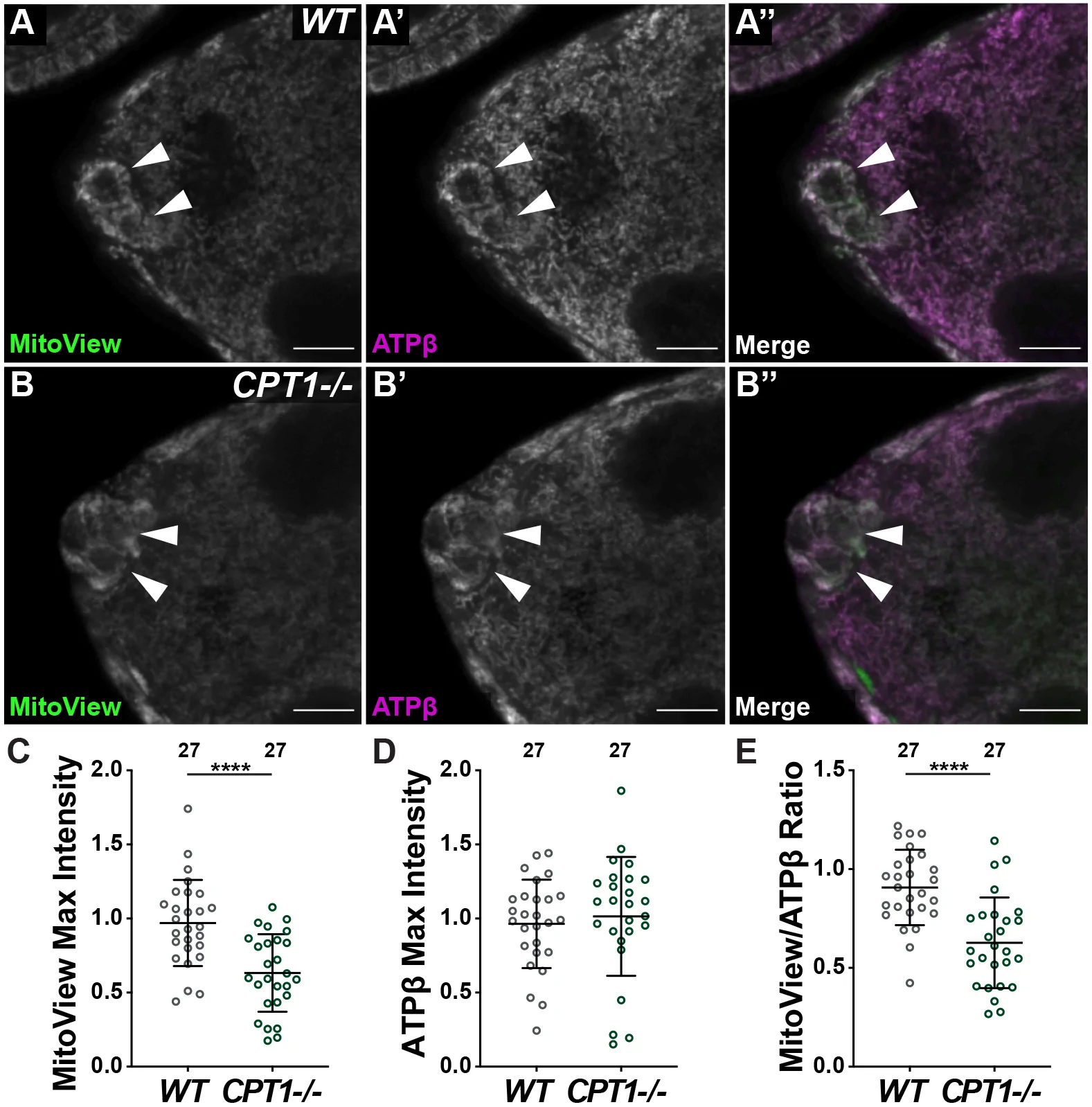
**CPT1 promotes mitochondrial function in the border cells.** (A-B″) Maximum projections of three confocal slices of S9 follicles stained for total mitochondria (ATPβ; magenta in merge) and oxidatively active mitochondria (MitoView; green in merge). White arrowheads indicate instances of colocalization between ATPβ and MitoView in the border cell (BC) cluster. Images are brightened by 50%. Black boxes have been placed behind panel and channel labels. Scale bars: 10 μm. (A-A″) Wild type (*yw*). (B-B″) *CPT1^−/−^* (*whd^1^/whd^1^*). (C-E) Quantification of maximum fluorescence intensity of MitoView (C), maximum fluorescence intensity of ATPβ (D) and ratio of MitoView to ATPβ (E) in the BC cluster for the indicated genotypes. *n*, number of clusters (listed at the top of each set of data); line indicates the mean; error bars indicate s.d. ns, *P*>0.05; ****P<0.0001 (unpaired *t*-test, two-tailed). Loss of CPT1 reduces oxidatively active mitochondria without reducing total mitochondria (A-E).

To explore the role of FA β-oxidation in BC migration, we assessed the roles of CPT1, a rate-limiting enzyme for FA β-oxidation that transports long-chain FAs into the mitochondria (Strub et al., 2008). We first analyzed the BC mitochondria of *CPT1* null mutants (Fig. 8A-B″). Loss of CPT1 lowers the polarization-dependent mitochondrial signal (Fig. 8C; *P*<0.0001) without altering the polarization-independent signal (Fig. 8D). Consequently, loss of CPT1 decreases the ratio of polarization-dependent to polarization-independent staining (Fig. 8E; *P*<0.0001), indicating a loss of mitochondrial membrane potential in the BC cluster. Thus, CPT1 is required for normal mitochondrial function in the BCs.

**Fig. 9.**
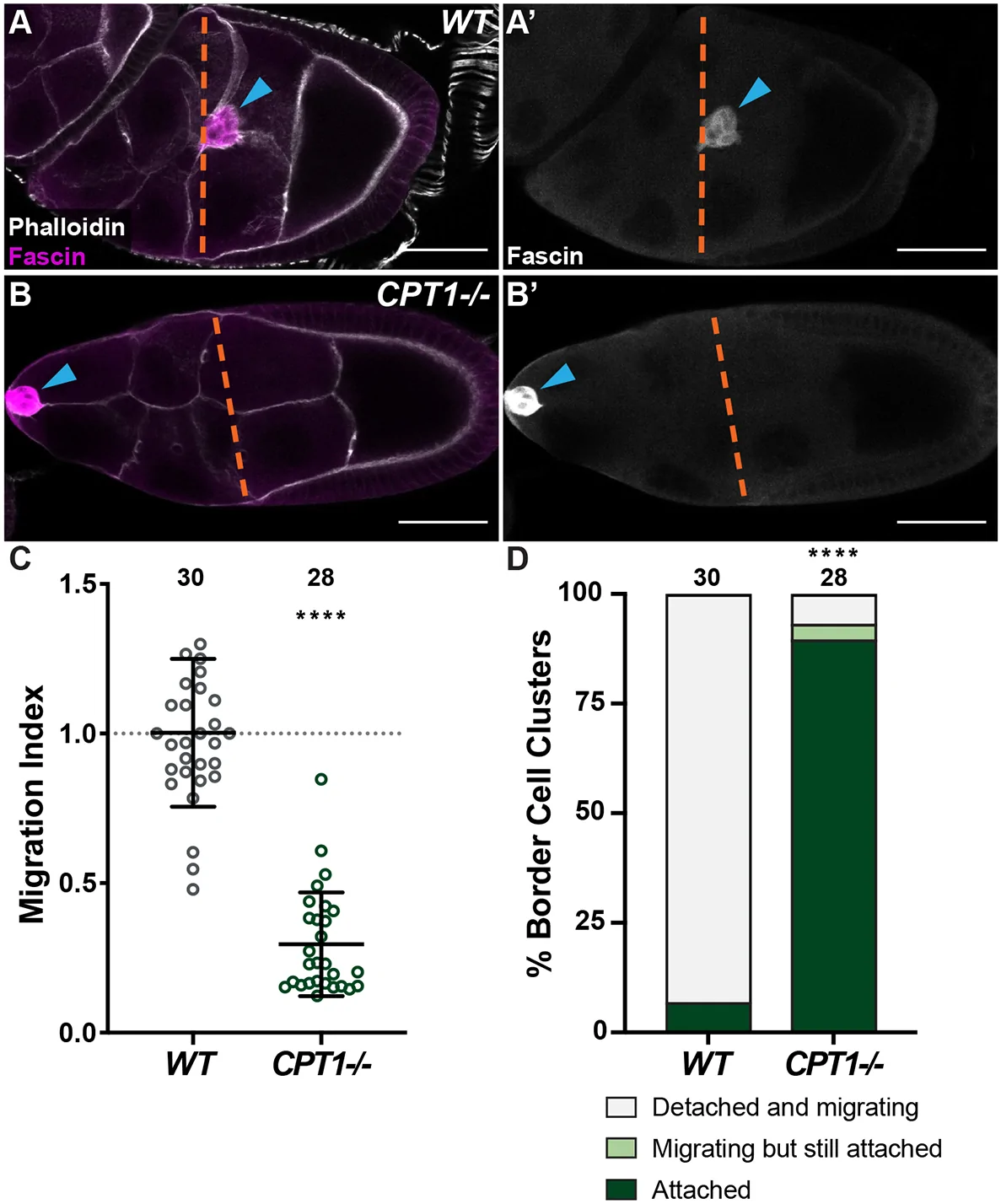
**CPT1 is required for border cell detachment and on-time migration.** (A-B′) Maximum projections of three confocal slices of S9 follicles stained for Fascin [border cells (BCs); magenta] and F-actin (phalloidin; white). Blue arrowheads indicate BC clusters. Orange dashed lines indicate the position of the outer follicle cells. Black boxes have been placed behind channel labels. Scale bars: 50 μm. (A,A′) Wild type (*yw*). (B,B′) *CPT1^−/−^* (*whd^1^/whd^1^*). (C) Graph of the migration index for the indicated genotypes. *n,* number of follicles (listed at the top of each set of data); dotted line indicates on-time BC migration; solid line indicates mean; error bars indicate s.d. ****P<0.0001 (unpaired *t*-test, two-tailed). (D) Quantification of BC detachment defects for the indicated genotypes. BC clusters categorized as detached and migrating (light gray), migrating but still attached (light green) or attached (dark green). *n,* number of clusters (listed at the top of each set of data); ****P<0.0001 (Pearson's Chi-squared test with Monte Carlo simulation). Loss of CPT1 delays BC migration (A-C) and severely blocks detachment from the follicular epithelium (D).

We next assessed the role of CPT1 in BC migration. Loss of CPT1 results in delayed BC migration (Fig. 9A-C; MI=0.295 compared to MI=1.002, *P*<0.0001) and, strikingly, results in 89.3% of BC clusters remaining attached to the anterior follicular epithelium, compared to 6.6% in the wild type (Fig. 9D; *P*<0.0001). Together, these findings strongly suggest that import of FAs into the mitochondria, including FAs released from LD stores by ATGL, is crucial for BC delamination and therefore, migration.

Finally, we sought to determine the cell-specific role of CPT1 during BC migration. RNAi knockdown of CPT1 in the BCs does not alter mitochondrial function in the BC cluster (Fig. S10). Whereas knockdown of CPT1 in the BCs delays their migration (Fig. S11A-D; MI=0.636) compared to the GAL4- (MI=0.985; *P*<0.0001) and RNAi-only (MI=0.852; *P*<0.0001) controls. Furthermore, BC knockdown of CPT1 resulted in 26% of BC clusters remaining attached to the anterior follicular epithelium during S9 (Fig. S11E), compared to 6% and 8.3% in the GAL4- (*P*<0.01) and RNAi-only (*P*<0.001) controls, respectively. These migration and detachment phenotypes are much less severe than those observed in the mutant, suggesting the RNAi knockdown may not sufficiently deplete CPT1. These results support that CPT1 and import of FAs into the mitochondria in the BCs facilitates delamination and on-time migration.

## DISCUSSION

**Fig. 10.**
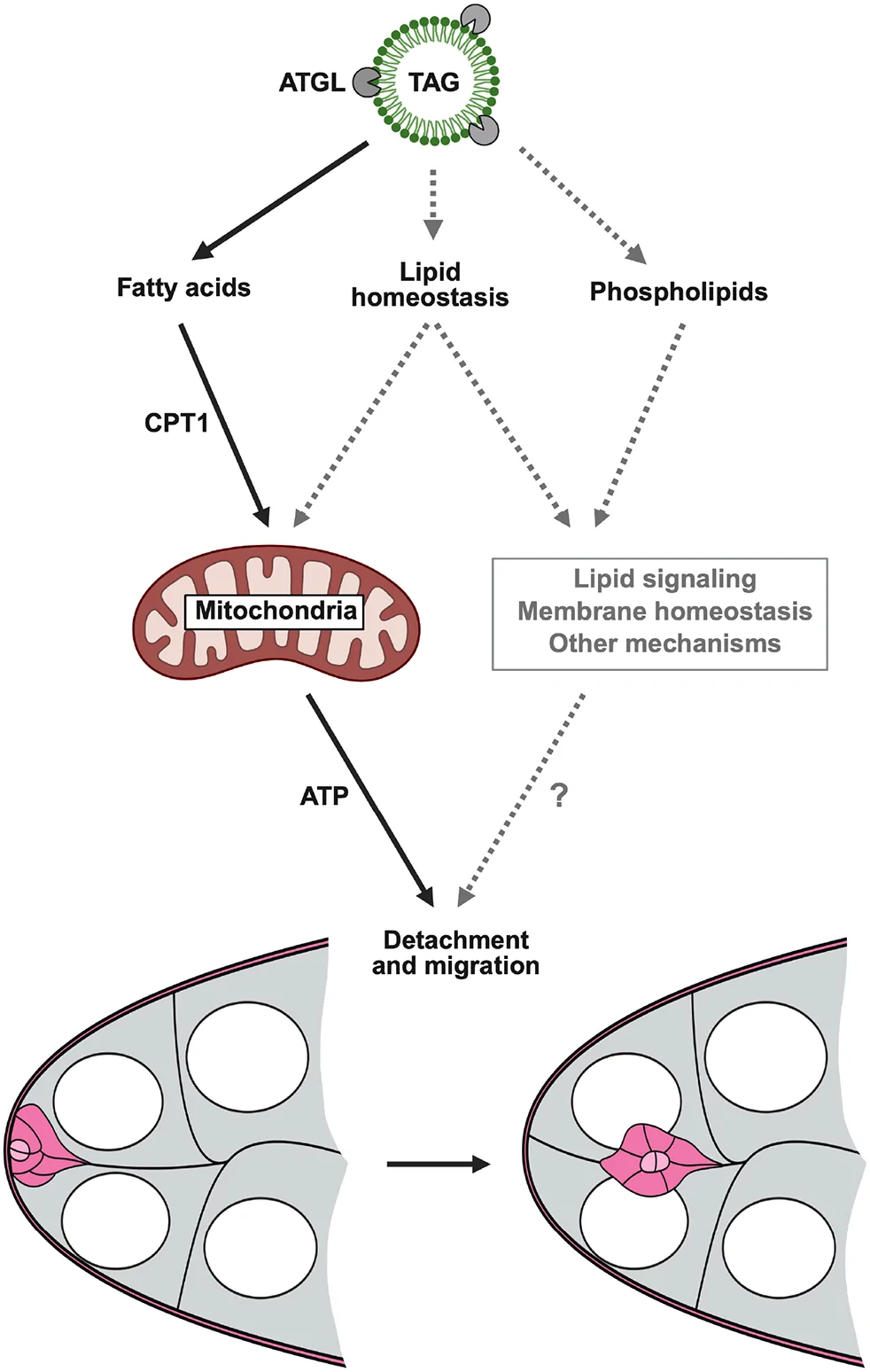
**Model for how ATGL-mediated lipolysis promotes border cell delamination and migration.** Schematic summarizing our findings. Lipid droplets (LDs; green) are present in the border cell (BC) cluster (pink). The lipase ATGL (gray notched circles) cleaves LD-stored triglycerides (TAGs) to generate free fatty acids, which undergo CPT1-dependent β-oxidation in mitochondria (brown) for ATP production (solid arrows). Other possible fates (dashed arrows) for ATGL-released lipids in the BCs include lipid homeostasis and phospholipid production, which in turn contribute to lipid signaling, membrane homeostasis and/or other cellular functions. Ultimately, ATGL-released lipids promote BC delamination and migration. Created with BioRender.com by Wipf, I., 2026. https://BioRender.com/ao2u3hy. This figure was sublicensed under CC-BY 4.0 terms.

Using *Drosophila* BC migration as a model, we provide the first evidence that regulated mobilization and utilization of lipids from LDs promotes *in vivo* delamination and collective migration (Fig. 10). BCs store neutral lipids in LDs prior to and during migration. These LDs undergo changes in size and number throughout migration and LDs exhibit polarity within the cluster. We identify ATGL as an important regulator of LD lipolysis in the BCs; loss of ATGL increases LD size within the BC cluster. ATGL is required in the BCs for both on-time migration and delamination. ATGL activity must be carefully regulated. Excess release of stored lipids could lead to lipotoxicity or cellular dysfunction, especially if those lipids are unavailable when needed (Obaseki et al., 2024). Indeed, BC overexpression of ATGL depletes LD stores within the cluster, impairs delamination and delays migration. However, evidence supports there is not generalized lipid dysfunction in the absence of ATGL, as ER morphology is maintained. Rather, ATGL regulates mitochondrial function. Loss of ATGL alters mitochondrial morphology and distribution, decreases mitochondrial membrane potential relative to organelle mass, and ROS accumulates in the mitochondria within the BC cluster. Evidence supports that these mitochondrial defects are due, in part, to decreased FA β-oxidation, as loss of CPT1, the enzyme that transports FAs into the mitochondria for β-oxidation (Houten et al., 2016), results in failed delamination, delayed migration, and altered mitochondrial function. Together these data lead to the model that ATGL-dependent LD lipolysis is tightly regulated during BC migration to, in part, ensure that FAs are available for mitochondrial β-oxidation to provide energy to fuel delamination and migration.

### Lipolysis facilitates BC migration

Mobilization and utilization of lipids from LDs is crucial for BC delamination and migration. Few LDs are observed in the non-migratory polar cells within the cluster, whereas LDs are abundant in the BCs throughout migration but are larger and fewer in number pre- and during migration than at the end of migration. LD size and number are regulated by LD biogenesis, fusion and utilization. Large LDs indicate older LDs or a cellular state promoting lipid storage, while smaller LDs indicate LD biogenesis or high lipid utilization, resulting in LD shrinkage (Walther and Farese, 2012; Welte, 2015; Olzmann and Carvalho, 2019). We speculate that biogenesis and utilization both occur during BC migration to maintain LD stores while fueling migration. Lipid needs shift at the end of migration, resulting in more numerous and smaller LDs – a change suggesting biogenesis and/or depletion of LD stores. Further, LD distribution exhibits polarity within the BC cluster, with fewer LDs in the front and one lateral side compared to the rear and other lateral side. As the leading BC is regularly replaced with a lateral cell (the prior leader becomes a lateral cell in the opposite direction; Prasad and Montell, 2007), the LD polarity may reflect LD depletion in both the leader and recently replaced leaders (lateral low).

Supporting the model that lipids from LDs are being utilized during BC migration, loss of the rate limiting enzyme in triglyceride lipolysis, ATGL, increases LD size but not number. The latter suggests that either small, newly formed LDs rapidly fuse with existing LDs or there is a feedback mechanism preventing LD biogenesis when lipids are not mobilized from LDs. Loss or BC knockdown of ATGL results in impaired delamination and delayed migration, revealing that this lipolytic enzyme is crucial for the initial invasion and collective migration of the BCs. Notably, RNAi knockdown of ATGL does not result in major LD changes; we speculate this is due to ATGL levels being reduced, but not absent, due to either the timing of or extent of knockdown. It is also possible that ATGL activity within other cells also regulates BC LDs. Finally, BC overexpression of ATGL depletes LDs, impairs delamination and delays migration. We postulate the migration defects are due to LD lipids being unavailable when needed. Thus, both loss of ATGL and overexpression of ATGL result in the same consequence: LD lipids cannot be released due to the lack of the required lipase or the lack of LDs, respectively. The similarity in the migration defects between too little and too much ATGL supports that ATGL levels, and likely activity, must be tightly regulated to ensure lipid utilization from LDs occurs at the right time, level and location for BC delamination and migration.

### How does ATGL promote delamination and migration?

As tight regulation of lipid storage and release from LDs is crucial for cellular functions (Obaseki et al., 2024), the roles of ATGL in BC migration are likely to be complex. For example, if ATGL activity maintains normal lipid metabolism, changes in ATGL levels will cause broad dysregulation. Indeed, metabolomic studies show dysregulation of multiple lipid species, including phospholipids, diacylglycerides, triglycerides and FAs when ATGL is lost or reduced (Chitraju et al., 2013; Salatzki et al., 2018; Hofbauer et al., 2021; Nazario-Yepiz et al., 2021; Oluwadare et al., 2022; Awad et al., 2024; Chao et al., 2024; Manceau et al., 2026). In *Drosophila* ovaries, ATGL loss alters specific phospholipid and triglyceride species (Giedt et al., 2023). General lipid dysregulation results in multi-organelle dysfunction, including the mitochondria and ER (Manceau et al., 2026). While we observe altered mitochondrial morphology and function, the ER appears morphologically normal when ATGL is lost. Further, the delamination and migration defects due to altered ATGL levels are not observed when other key LD metabolic regulators are lost. These data lead us to postulate that the migration defects due to altered ATGL levels are not due to broad lipid dysregulation.

As ATGL regulates the levels of specific phospholipid species (Chitraju et al., 2013; Salatzki et al., 2018; Hofbauer et al., 2021; Oluwadare et al., 2022; Awad et al., 2024; Chao et al., 2024; Manceau et al., 2026), another possible function of ATGL is to regulate membrane composition, stiffness and/or dynamics. Altered phospholipid composition is observed in cancer, where certain compositions favor proliferation and others favor migration (Braig et al., 2015; Abdelbaset-Ismail et al., 2019; Szlasa et al., 2020). Altered membrane composition also impacts receptor function and signaling (Dawaliby et al., 2016; Izard and Brown, 2016; Abdelbaset-Ismail et al., 2019). Thus, one untested potential role of ATGL is in regulating membrane phospholipids to facilitate BC migration.

Another potential role of ATGL is to regulate PG signaling. In multiple human cell contexts (Dichlberger et al., 2014; Schlager et al., 2015) and during S10B of *Drosophila* oogenesis (Giedt et al., 2023), ATGL releases AA from LD triglycerides to drive PG synthesis. Our data indicate that this is not the case during BC migration, as ATGL does not exhibit a dominant genetic interaction with dCOX1.

Conversely, our data support the model that the defects in delamination and migration when ATGL levels are perturbed are due, in part, to effects on mitochondria. Loss of ATGL results in clustered, elongated and, perhaps, increased numbers of mitochondria in the BCs. These findings are consistent with increased mitochondrial fusion or increased mitochondrial biogenesis, or both. Mitochondrial fission is crucial for BC migration, as impairing fission by loss of Drp1 or overexpression of mitofusins delays and impairs the completion of migration (Qu et al., 2022). The study suggests, through *in vitro* and whole-ovary analyses, that the migration defects are due to reduced mitochondrial ATP production (Qu et al., 2022). Supporting similar mitochondrial defects arise due to loss of ATGL, while there appears to be increased mitochondria, there is not a proportional increase in membrane potential, indicating reduced membrane potential per unit of mitochondria – a finding indicative of mitochondrial dysfunction. Further, loss of ATGL triggers mitochondrial ROS production. This mitochondrial dysfunction could be the result of numerous functions of ATGL. Altering ATGL levels could cause broad lipid dysregulation (Chitraju et al., 2013; Salatzki et al., 2018; Hofbauer et al., 2021; Nazario-Yepiz et al., 2021; Oluwadare et al., 2022; Awad et al., 2024; Chao et al., 2024; Manceau et al., 2026), and thereby impair mitochondrial function; we do not favor this possibility. Alternatively, loss of ATGL could alter the levels of phospholipid species, and mitochondrial function is sensitive to changes in phospholipids (Becker et al., 2013; Vance, 2015; Yang et al., 2018). Further studies are needed to explore these possibilities.

Supporting the theory that the mitochondrial dysfunction in the *ATGL* mutant is due, in part, to altered FA β-oxidation, loss of CPT1 results in BC delamination defects and mitochondrial dysfunction as in *ATGL* mutants. The delamination failure is more severe in *CPT1* mutants than in *ATGL* mutants, suggesting that other sources of FAs, besides those released from LDs by ATGL, are important for BC invasion and migration. Together, the data support the model that ATGL-released FAs from LDs are used, in part, for β-oxidation within the mitochondria to produce the energy necessary for the delamination and on-time migration of the BCs. Supporting this ATGL function is conserved, in skeletal muscle, ATGL-mediated lipolysis is essential for mitochondria function, as mutating or inhibiting ATGL decreases mitochondrial-LD interaction and disrupts mitochondria respiration and network connectivity (Gemmink et al., 2026).

### LDs, ATGL and β-oxidation – conserved regulators of invasion and migration

The functions of ATGL, LDs and mitochondrial β-oxidation in invasive migration are likely conserved across developmental contexts and in cancer metastasis. Indeed, mouse migrating neural crest cells accumulate LDs, although the function is unknown (Patel et al., 2015). Most evidence supporting roles for LDs, lipolysis and β-oxidation in migration come from *in vitro* studies on cancer cells and correlative studies on human patient samples. Metastatic breast cancer cells exhibit higher LD accumulation compared to non-metastatic cells; knocking down ATGL or inhibiting FA β-oxidation reduces 2D migration and 3D invasion (Wang et al., 2017b; Andolino et al., 2025). Similar findings have been observed in pancreatic cancer cell lines (Rozeveld et al., 2020). Supporting the idea that LDs contribute to cancer progression in humans, breast cancer lung metastases from patients show LD accumulation (Andolino et al., 2025) and LD density positively correlates with higher Gleason scores in prostate cancer (Nardi et al., 2019). Thus, LDs have emerged as mediators of cancer migration, invasion and metastasis, and are, thus, biomarkers of aggressive cancer and poor patient outcomes.

### Conclusions

Together our results reveal that tight regulation of ATGL-dependent release of lipids from LD stores within the BCs is crucial for their delamination and on-time migration. Evidence supports that ATGL-released FAs are used for β-oxidation. However, it remains possible that other ATGL-regulated lipids also promote BC delamination and migration. Given that LDs, ATGL and β-oxidation have emerged as key drivers of cancer cell migration, invasion and metastasis, we speculate this energy production pathway is widely used across migratory contexts.

### Study limitations

While the data support that tight regulation of the LD lipase ATGL is necessary for on-time delamination and migration of the *Drosophila* BCs, the underlying mechanisms remain to be fully elucidated. Evidence supports the theory that one mechanism is regulating mitochondrial function, because the mitochondria are larger and more prevalent, but exhibit reduced membrane potential relative to organelle mass and increased ROS when ATGL is lost; these findings are consistent with a reduction in FA β-oxidation. Adding support to this model, loss of CPT1, a mitochondrial FA transporter, results in both delamination defects and mitochondrial phenotypes. Future studies are needed to fully test this model and explore alternative models for ATGL function. Additionally, how LDs dynamically change in number, in polarity within the cluster, and in formation and utilization during migration remain unknown.

## MATERIALS AND METHODS

### Reagents and resources

See Table S1 for detailed information on the reagents used in these studies, Table S2 for the specific genotypes used in each figure panel and Table S3 for all raw data reported in this study.

### Fly stocks

Fly stocks were maintained on Bloomington standard fly food (cornmeal/agar/yeast food) at 22°C except where noted. The following stocks were used: *y^1^w^1^* [Bloomington Drosophila Stock Center (BDSC), #1495], *slbo GAL4* (BDSC, #58435), *bmm^1^* (BDSC, #98123; Gronke et al., 2005), *puml^KG01597^* (BDSC, #13732), *Hsl^EY20067^* (BDSC, #22358), *plin1^1^* (Beller et al., 2010), *plin1^2^* (Beller et al., 2010), *Lsd-2^KG00149^* (Teixeira et al., 2003), *Jabba^DL^* (Li et al., 2012), *Jabba^z101^* (Li et al., 2012), *dCOX1/pxt^f01000^* (Harvard Exelixis Collection; Thibault et al., 2004), *slbo GAL4* (BDSC, #58435), *UAS-bmm RNAi* (BDSC, #25926), *UAS-bmm* (BDSC, #76600), *whd^1^* (BDSC, #441) and *UAS-whd RNAi* (BDSC, #34066). Expression of the RNAi lines for follicle analyses was achieved by crossing to *slbo GAL4*, maintaining the fly crosses at room temperature (22°C) and maintaining progeny for BC analyses at 29°C for 4-5 days. Expression of other UAS lines (e.g. *UAS-bmm*) for follicle analyses was achieved by crossing to *slbo GAL4*, maintaining the fly crosses at room temperature and maintaining progeny for BC analyses at 25°C for 4-5 days. Before immunofluorescence staining, newly eclosed flies were fed wet yeast paste every day for 4-5 days.

### Immunofluorescence

*Drosophila* ovaries (5-8 pairs per sample) were dissected in room temperature Grace's insect medium (Corning). Ovaries were fixed for 10 min with 4% paraformaldehyde diluted in Grace's medium. Samples were washed six times for 10 min each at room temperature in antibody wash [1×phosphate-buffered saline (PBS), 0.1% Triton X and 0.1% bovine serum albumin]. The following primary antibodies were used: mouse anti-Fascin 1:50 (sn7c, Cooley, L., AB_528239; Cant et al., 1994), mouse anti-ATP-β 1:250 (ab14730; Abcam), mouse anti-Calnexin 99A 1:25 (Cnx99A 6-2-1, AB_2722011; Riedel et al., 2016) and mouse anti-Fasciclin III 1:50 (7G10, AB_528238; Patel et al., 1987). The anti-Fascin, anti-Calnexin 99A and anti-Fasciclin III primary antibodies were obtained from the Developmental Studies Hybridoma Bank (DSHB), which was developed under the auspices of the National Institute of Child Health and Human Development and maintained by the Department of Biology, University of Iowa (Iowa City, IA). Primary antibodies were diluted in antibody wash and incubated overnight at 4°C. The samples were washed six times for 10 min each at room temperature in antibody wash. Samples were then incubated with fluorescent secondary antibodies diluted 1:250 in antibody wash. Secondary antibodies used were goat anti-mouse Alexa Fluor 488 (AB_2534069; ThermoFisher Scientific) and goat anti-mouse Alexa Fluor 568 (AB_2534072; ThermoFisher Scientific). Alexa Fluor 568- or Alexa Fluor 647-conjugated Phalloidin (A12380 and A22287; Thermo Fischer Scientific) diluted 1:250 was included in both primary and secondary antibody incubations. To stain for LDs, following antibody staining, samples were incubated with BODIPY 493/503 (4,4-difluoro-1,3,5,7,8-pentamethyl-4-bora-3a,4a-diaza-s-indacene; Invitrogen, D3922) at a concentration of 1:100 in 1×PBS for 20 min at room temperature. After LD staining, 4′,6-diamidino-2-phenylidole (DAPI; 5 mg/ml; D3571; Thermo Fischer Scientific) staining was performed at a concentration of 1:5000 in 1×PBS for 10 min at room temperature. Finally, samples were rinsed three times in 1×PBS and mounted on coverslips in 1 mg/ml phenylenediamine in 50% glycerol at pH 9 (Platt and Michael, 1983).

### Mitochondrial membrane potential and superoxide production staining

To assess mitochondrial membrane potential, freshly dissected ovaries were incubated in S9 medium containing MitoView Fix 640 (200 nM, Biotium) for 90 min at room temperature (Prasad and Montell, 2007). S9 media consists of Schneider's medium (Sigma-Aldrich), 0.6× penicillin/streptomyocin (Life Technologies, SCR_008817), 0.2 mg/ml insulin (Sigma-Aldrich, SCR_008988) and 15% fetal bovine serum (Atlanta Biologicals). After MitoView staining, samples were rinsed twice in 1×PBS, then fixed and stained following the immunofluorescence protocol described above.

To assess mitochondrial superoxide production, freshly dissected ovaries were incubated in Grace's medium containing MitoSOX Red (5 μM, Invitrogen) for 15 min at room temperature. After MitoSOX staining, samples were rinsed twice in 1×PBS and fixed in 4% paraformaldehyde for 10 min. After fixation, samples were rinsed three times in 1×PBS and stained with DAPI (1:5000) for 10 min. Samples were then rinsed three more times in 1×PBS and mounted on coverslips in 1 mg/ml phenylenediamine in 50% glycerol at pH 9 (Platt and Michael, 1983).

### Image acquisition and processing

Microscope images for fixed and stained *Drosophila* follicles were taken using LAS-X software (SCR_013673) on a Leica DMi8 Stellaris within the Roy J. Carver Center for Imaging (RRID:SCR_028228) using a HCPLAPO CS2 20×/0.75 dry or HCPL APO CS2 63×/1.4 oil, or Zen software (SCR_013672) on Zeiss 700 LSM mounted on an Axio Observer.Z1 using a Plan-Apochromat 20×/0.8 M27 or EC-Plan_Neo_Fluar 40×/1.3 oil (Zeiss). S9 follicles were identified by their size (∼150 μm-250 μm) and morphology, including the location of the outer follicle cells and the BC cluster. The beginning of S10A was defined as when the anterior most outer follicle cells reached the nurse cell-oocyte boundary and flattened. Maximum projections, merge, rotation, cropping and confocal image stack movie generation were performed using ImageJ software (FIJI, RRID: SCR 002285; Abramoff et al., 2004). Images were brightened as indicated in the figure legends to improve visualization. Figures were made using Illustrator (Adobe, RRID: SCR 010279).

### Quantification of BC migration, detachment and cluster morphology

Quantification of the migration index (MI) was performed as described previously (Fox et al., 2020; Lamb et al., 2020; Mellentine et al., 2023). Briefly, measurements of S9 follicles were performed using ImageJ software (Abramoff et al., 2004) on *z*-stacks of follicles stained for DAPI, Fascin and phalloidin. A line segment was used to measure the distance in microns from the anterior end of the follicle to the leading edge of the BC cluster; this was defined as the BC distance. Another line segment was used to measure the distance from the anterior end of the follicle to the anterior end of the outer follicle cells: this was defined as the outer follicle cell distance. The entire follicle length was also measured along the anterior-posterior axis. The MI was calculated by dividing the BC distance by the follicle cell distance. The length of the BC cluster was determined by measuring the distance from the front to the rear of the BC cluster. The width of the BC cluster was determined by measuring the distance from one lateral side of the cluster to the other. The height of the BC cluster was determined by measuring the depth of the cluster (i.e. the number of confocal slices spanned by the cluster) and multiplying by the step size of the *z*-stack. The volume of the BC cluster was quantified using the Fascin signal and the 3D surface objects tool of Imaris (version 10.2; Bitplane). Attached clusters were not included in cluster morphology analyses. Data compilations and calculations were performed in Excel (Microsoft, RRID: SCR 016137), and graphs were generated and statistical analyses performed using Prism (GraphPad Software, RRID: SCR 002798).

Z-stacks projection images of S9 follicles were used to analyze the position of the BC cluster for detachment defects. S9 follicles stained for DAPI, Fascin and phalloidin were classified into three categories: those with BC clusters that are (1) fully detached and migrating, (2) migrating but have a tail that remains attached to the anterior follicular epithelium or (3) attached to the anterior follicular epithelium. Only mid- to late-S9 follicles were scored; early S9 follicles were not included in delamination quantifications. Data compilations and calculations were performed in Excel, graphs were generated using Prism and Pearson's Chi-squared analyses were performed using RStudio [4.5.2 (2025-10-31)].

### Lipid quantification

S9 follicles were stained and imaged on a Leica DMi8 Stellaris, as described above. The resulting z-stacks were converted to Imaris files (.ims). To analyze LDs specifically in the BC cluster, the 3D surface objects tool of Imaris (version 10.2; Bitplane) was used to create a region of interest from the Fascin signal. This approach was used to create a Fascin-positive region of interest for the BC cluster, called the BC surface. To restrict LD signal to the BC cluster, the BODIPY 493/503 signal was masked to the BC surface. Following masking, the abundance and volume of LDs within the BC cluster was quantified using the 3D surface objects tool of Imaris. LD surfaces were generated using automated thresholding (background subtraction) performed with local contrast. To resolve closely apposed objects, the split touching objects (region growing) function was enabled with a seed point diameter of 0.6 μm, followed by a morphological split. Seed points were filtered by applying a quality threshold above an automated cutoff. The analysis settings were kept constant between genotypes. The same process was used to analyze LDs specifically in the polar cells, except Fasciclin III was used to define the region of interest instead of Fascin. Following rendering, LD quantifications were exported as Microsoft Excel files. Each data point represents the averaged value within one BC cluster. Data were compiled in and calculations were carried out in Excel, and graphs were generated and statistical analyses performed using Prism.

To analyze the distribution of LDs within the BC cluster, the Fascin-positive BC surface was divided into four regions using the cut surface tool of Imaris: a back or anterior region, two lateral side regions, and a front or posterior region. LD parameters (i.e. abundance and volume) were then analyzed in each of these defined regions using the 3D surface objects tool of Imaris as described above. The lateral sides within a cluster were defined as lateral high and lateral low, based on the number of LDs; two clusters had the same number of LDs in their lateral sides, so for these clusters the mean LD volume was used to define lateral high and low.

### Mitochondrial quantifications

To quantify mitochondrial membrane potential, MitoView and ATP-β intensities were measured in ImageJ from single confocal slices of immunofluorescence images of fixed S9 follicles. The ‘straight line’ function was used to draw lines between 4 and 7 µm at three different locations within the BC cluster, and the highest fluorescence intensity value was measured for both MitoView and ATP-β. To calculate the MitoView to ATP-β staining ratio, the average value was calculated for the three line segments for each BC cluster, then the MitoView value was divided by the ATP-β value, and the average was normalized to the overall wild-type average. Data were compiled in and calculations were performed in Excel, and graphs were generated and statistical analyses performed using Prism.

To quantify mitochondrial superoxide production, MitoSOX intensity was measured in ImageJ from single confocal slices of fixed S9 follicles. The ‘straight line’ function was used to draw lines between 4 and 7 µm at three different locations within the BC cluster, and the highest fluorescence intensity value was measured for MitoSOX. Lines between 4 and 7 µm were also used to measure the background intensity value outside of the follicle, which was subtracted. The average value was then calculated for the three line segments and the average was normalized to the overall wild-type average. Data were compiled in and calculations were performed in Excel, and graphs were generated and statistical analyses performed using Prism.

## Supplementary Material



10.1242/develop.205486_sup1 Supplementary information

Table S1. Reagents Table. List of reagents used in the study.

Table S2. Genotypes by figure. List of genotypes shown in each figure panel.

Table S3. Raw data. Raw data used for all quantifications presented in both the primary and supplemental figures.
