# Supplementary material for: ATGL-mediated lipid droplet lipolysis promotes collective cell migration in Drosophila
Source: Development. 2026 Aug 20;153(17):dev205486. doi: 10.1242/dev.205486 (PMC13539429; doi:10.1242/dev.205486)
Supplement: Supplementary file 1 [file develop-153-205486-s1.pdf]

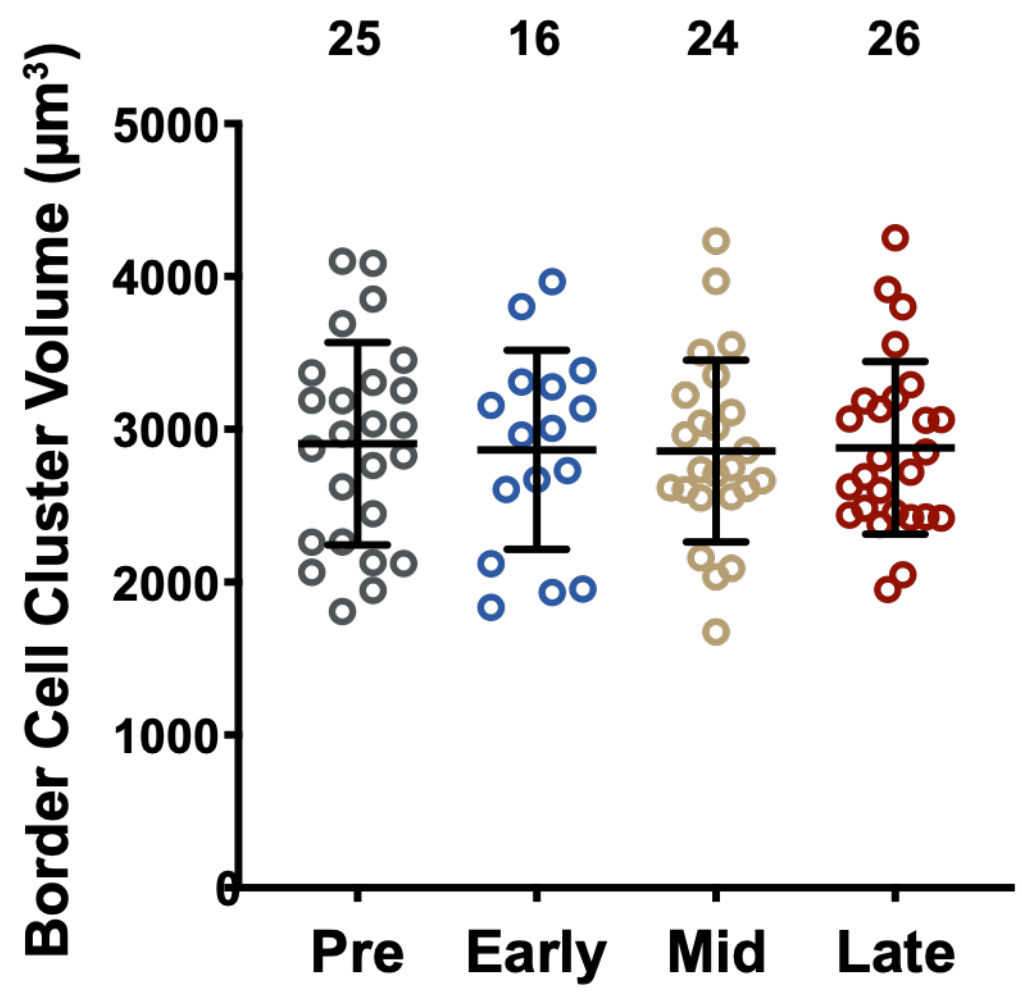

Fig. S1. Border cell cluster volume is unchanged throughout migration. Quantification of the *wild-type* (*yw*) border cell cluster volume at the indicated stage of migration. Circle = individual border cell cluster, n = number of border cell clusters, line = mean, error bars = SD, and ns > 0.05, one-way analysis of variance (ANOVA) with Tukey multiple comparison test. In *wild-type* (*yw*) flies, the size of the border cell cluster (~3000 μm^3) does not change throughout migration.

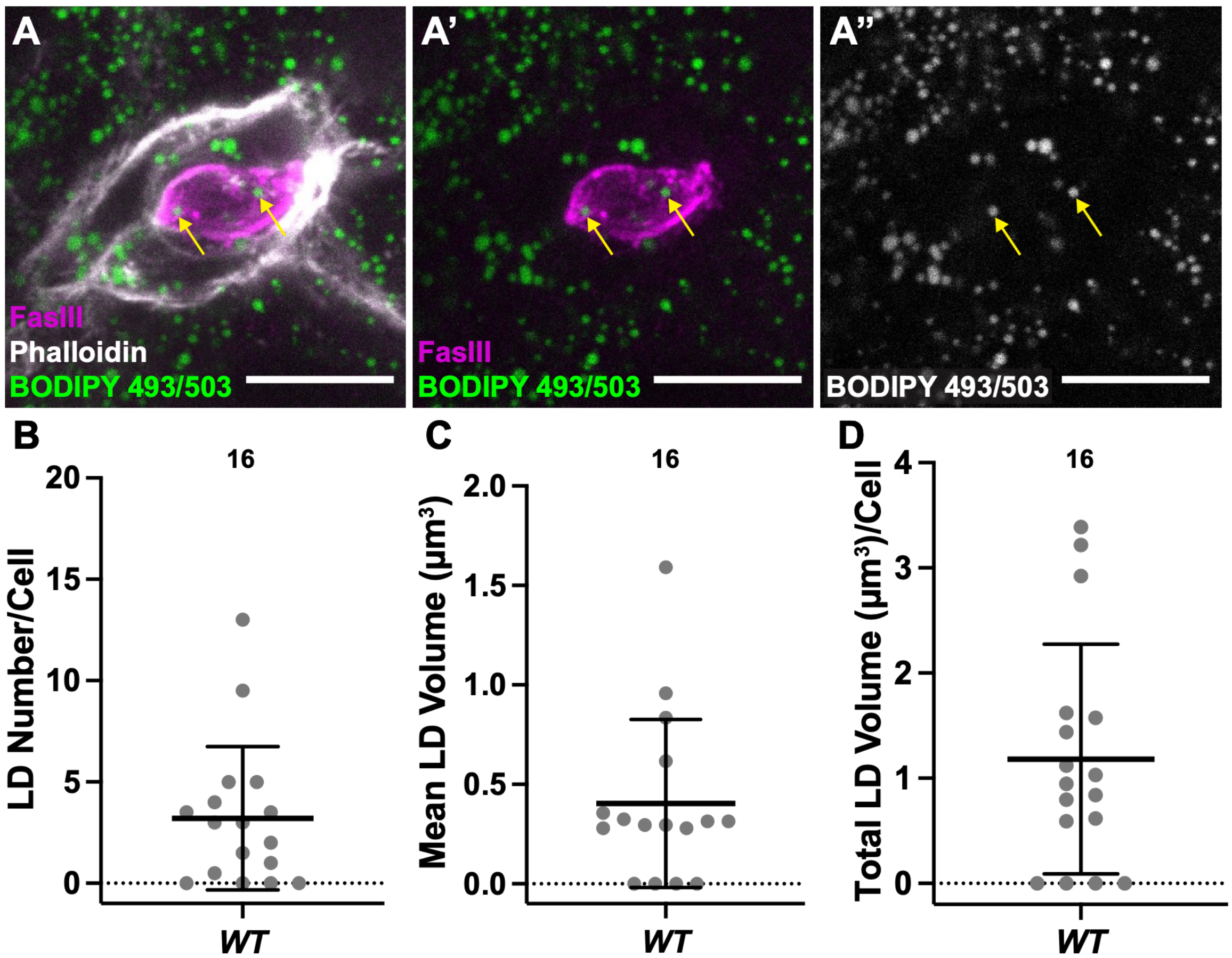

Fig. S2. Lipid droplets (LDs) are scarce but present in the polar cells during border cell migration. (A-A"). Maximum projections of 3 confocal slices of wild-type (*yw*) Stage 9 follicles stained for Fasciclin III (FasIII) in magenta, F-actin (phalloidin) in white, and LDs (BODIPY 493/503) in green. Yellow arrows indicate examples of LDs in the polar cells. LD signal brightness was increased by 70% to improve image clarity. Black boxes placed behind panel and channel labels to increase legibility. Scale bars = 10 µm. **(B-D).** Quantification of LD number **(B)**, mean LD volume **(C)**, and total LD volume **(D)** in the polar cells. Circle = individual border cell cluster, n = number of border cell clusters, line = mean, error bars = SD. LDs are often (but not always) present within the polar cells during border cell migration.

Development • Supplementary information

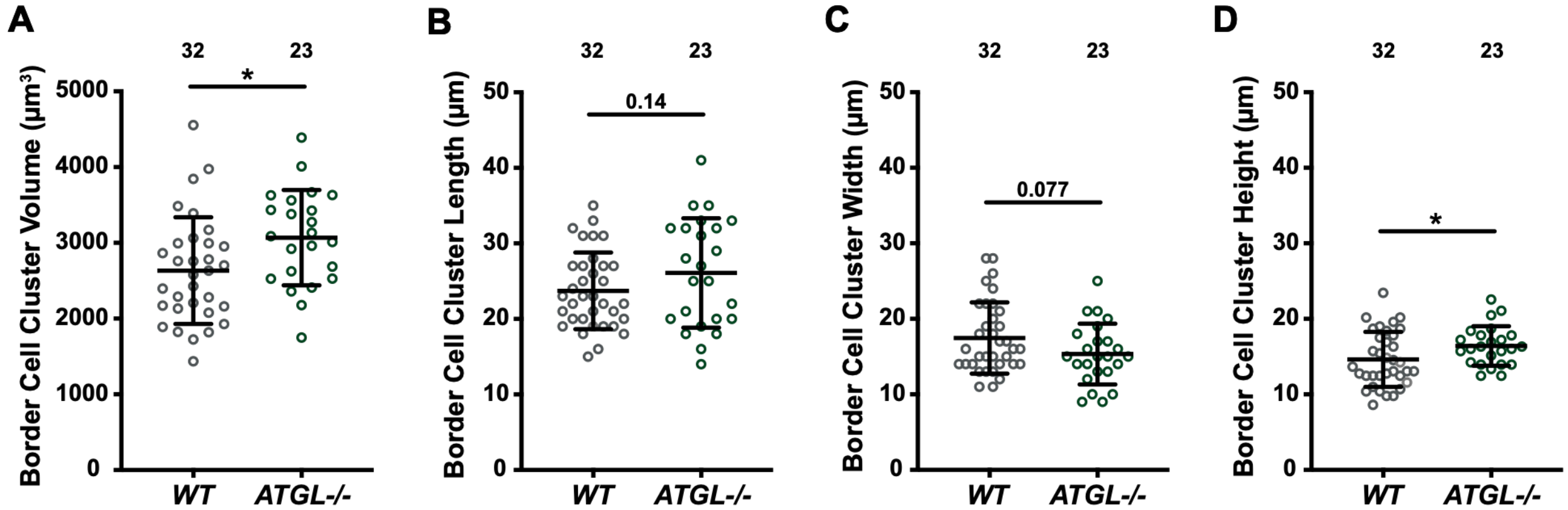

Fig. S3. ATGL regulates border cell cluster morphology. (A-D). Quantification of the border cell cluster volume **(A)**, length **(B)**, width **(C)**, and height **(D)** in *wild-type* (*yw*) and *ATGL* (*bmm*[1]/*bmm*[1]) mutants. Circle = individual border cell cluster, n = number of border cell clusters, line = mean, error bars = SD, and ns > 0.05, * $p < 0.05$, unpaired *t*-test, two-tailed. **(A-D).** Loss of ATGL significantly increases the volume **(A)** and height **(D)** of the border cell cluster.

Development • Supplementary information

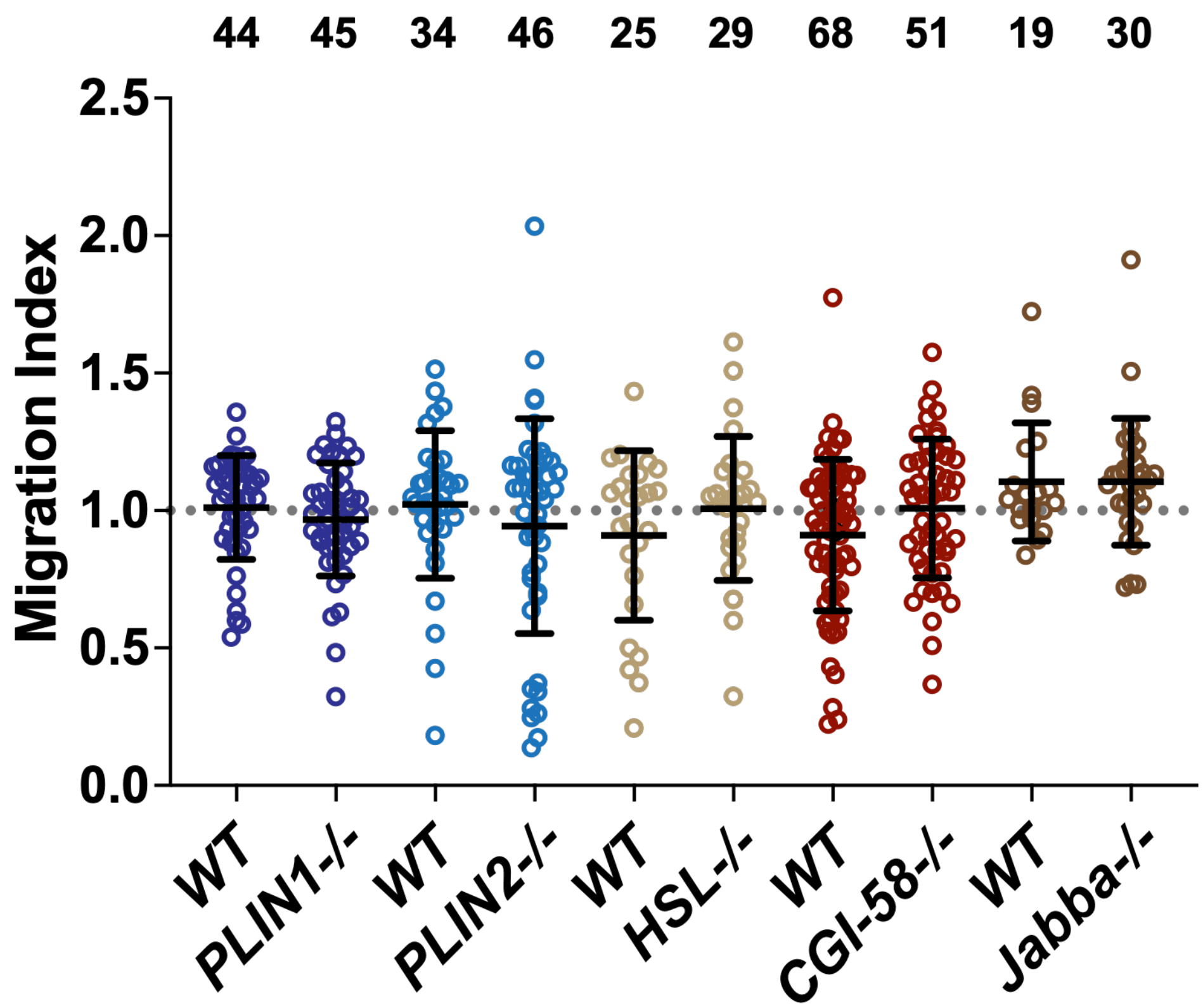

Fig. S4. Several lipid droplet proteins are not required for on-time border cell migration. Graph of migration index for the indicated genotypes paired with their *wild-type* (*yw)* controls: *PLIN1-/-* (*plin1^{1}/plin1^{2}*; purple), *PLIN2-/-* (*Lsd-2^{KG00149}/Lsd-2^{KG00149}*; blue), *HSL-/-* (*Hsl^{EY20067}/ Hsl^{EY20067}*; tan), *CGI-58-/-* (*puml^{KG01597}/puml^{KG01597}*; red), *Jabba-/-* (*Jabba^{z101}/Jabba^{z101}* and *Jabba^{DL}/Jabba^{DL}* combined; brown). Circle = individual border cell cluster, n = number of border cell clusters. The dotted line = on-time border cell migration, solid black lines = means, error bars = SD, and ns = $p > 0.05$, unpaired *t*-test, two-tailed. The lipid droplet-associated proteins PLIN1, PLIN2, HSL, CGI-58, and Jabba are not required for on-time border cell migration.

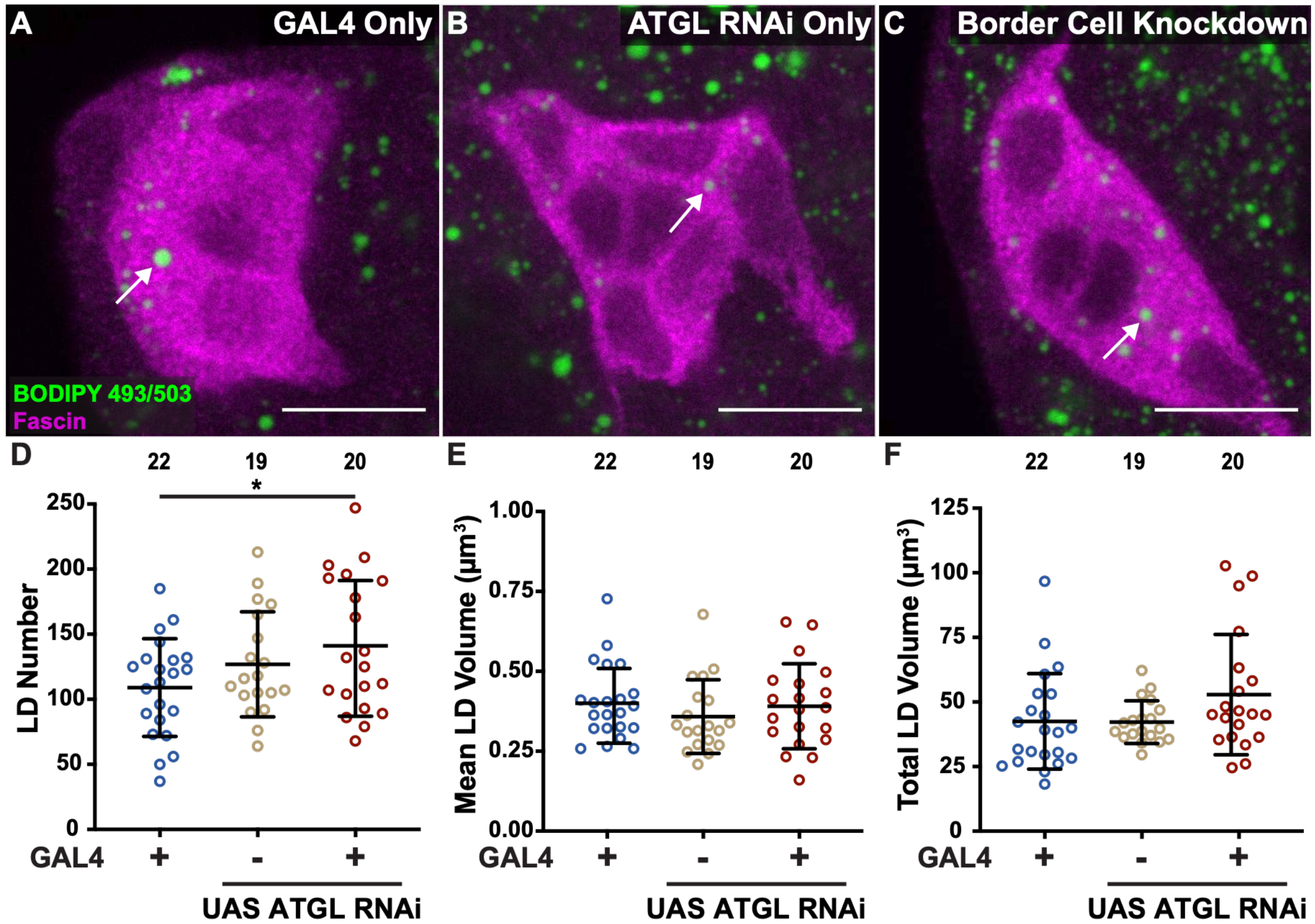

Fig. S5. Knockdown of ATGL in the border cells increases LD number but does not alter LD volume. (A-C). Maximum projections of 3 confocal slices of S9 follicles stained for LDs (BODIPY 493/503) in green and Fascin (border cells) in magenta. White arrows indicate examples of LDs in the border cell cluster. Images brightened by 50% to increase clarity. Black boxes placed behind panel and channel labels to increase legibility. Scale bars = 10 µm. **(A)** GAL4 control (*slbo GAL4/+*). **(B)** ATGL RNAi control (*UAS bmm RNAi/+*). **(C)** Border cell knockdown of ATGL (*slbo GAL4/UAS bmm RNAi*). **(D-F).** Quantification of LD number **(D)**, mean LD volume **(E)**, and total lipid volume **(F)** in the border cell clusters for the indicated genotypes. Circle = individual border cell cluster, n = number of border cell clusters, line = mean, error bars = SD, * $p<0.05$, one-way ANOVA with Tukey multiple comparison test. Border cell knockdown of ATGL does not alter LD volume **(E, F)** but increases LD number in the border cells **(D)** compared to the GAL4 only control.

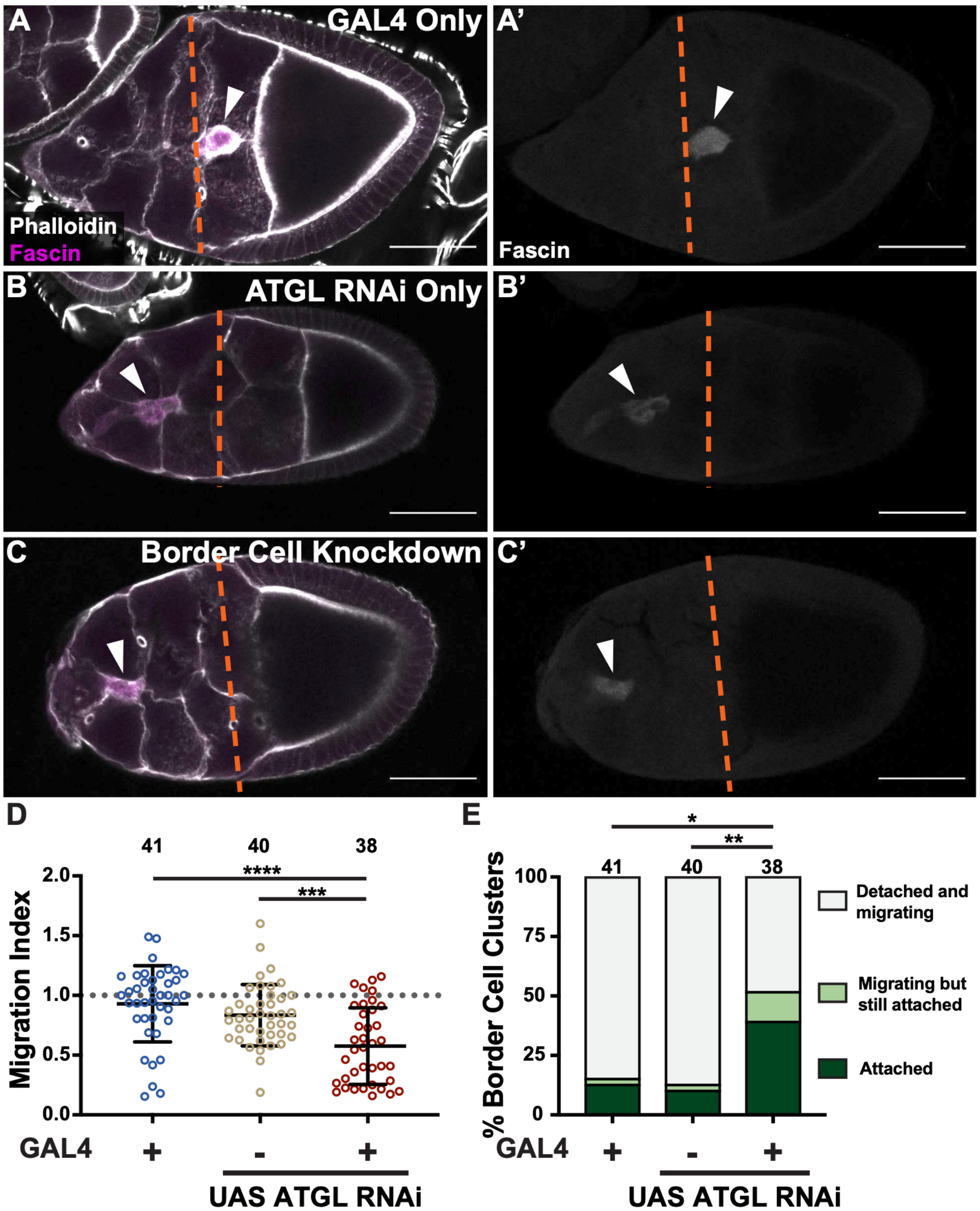

Fig. S6. ATGL is required in the border cells for detachment and on-time migration. (A-C). Maximum projections of 3 confocal slices of S9 follicles stained for Fascin (border cells) in magenta and F-actin (Phalloidin) in white. White arrowheads indicate the border cell cluster, and orange dashed lines indicate the anterior edge of the outer follicle cells.

Development • Supplementary information

Images brightened by 50% to increase clarity. Black boxes placed behind panel and channel labels to increase legibility. Scale bars = 50 μm. **(A, A')** GAL4 control (*slbo GAL4/+*). **(B, B')** ATGL RNAi control (*UAS bmm RNAi/+*). **(C, C')** Border cell knockdown of ATGL (*slbo GAL4/ UAS bmm RNAi*). **(D).** Graph of migration index for the indicated genotypes. Circle = individual border cell cluster, n = number of border cell clusters. The dotted line = on-time border cell migration, solid black lines = means, error bars = SD, and ns > 0.05, *** p < 0.001, **** p < 0.0001, one-way ANOVA with Tukey multiple comparison test. **(E).** Quantification of border cell detachment defects for the indicated genotypes. Border cell clusters categorized as detached and migrating (light grey), migrating but still attached (light green), or attached (dark green). *n* = number of border cell clusters, and ns > 0.05, * p < 0.05, ** p < 0.01, Pearson's Chi-squared test with Monte Carlo simulation. Border cell knockdown of ATGL delays border cell migration during S9 **(C, D)** and impairs detachment of the border cell cluster from the follicular epithelium **(E)**.

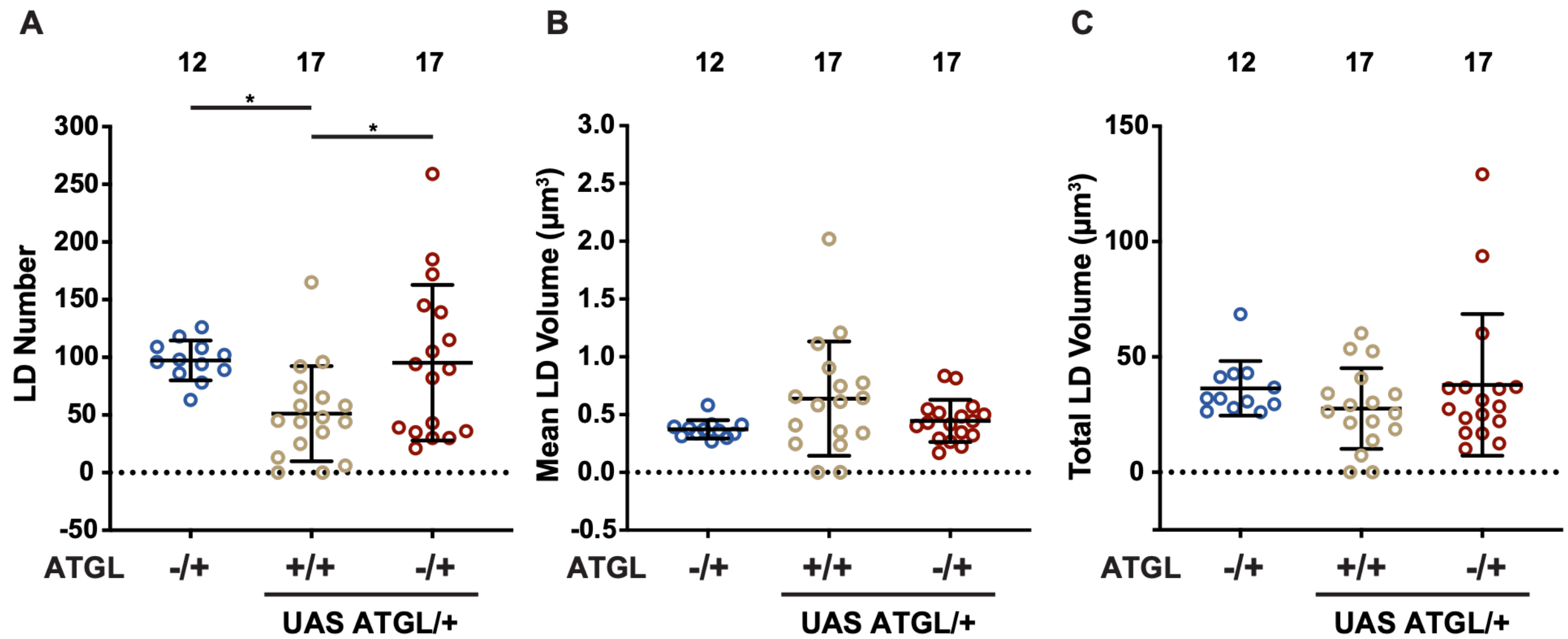

Fig. S7. ATGL heterozygosity rescues LD numbers in UAS ATGL border cells. (A-C). Quantification of LD number **(A)**, mean LD volume **(B)**, and total LD volume **(C)** in the border cell clusters for the following genotypes: ATGL heterozygote (*ATGL/+*), UAS ATGL (*UAS ATGL/+*), UAS ATGL in ATGL heterozygous background (*ATGL/UAS ATGL*). Circle = individual border cell cluster, n = number of border cell clusters, line = mean, error bars = SD, and ns > 0.05, * $p < 0.05$, one-way ANOVA with Tukey multiple comparison test. **(A-C).** UAS ATGL border cells have reduced LD numbers compared to ATGL heterozygotes **(A)**; however, reducing ATGL dosage (ATGL heterozygosity) restores LD numbers in UAS ATGL border cells to levels comparable to ATGL heterozygotes **(A)**. Meanwhile, there are no significant differences in mean LD volume **(B)** or total LD volume **(C)** between genotypes.

Development • Supplementary information

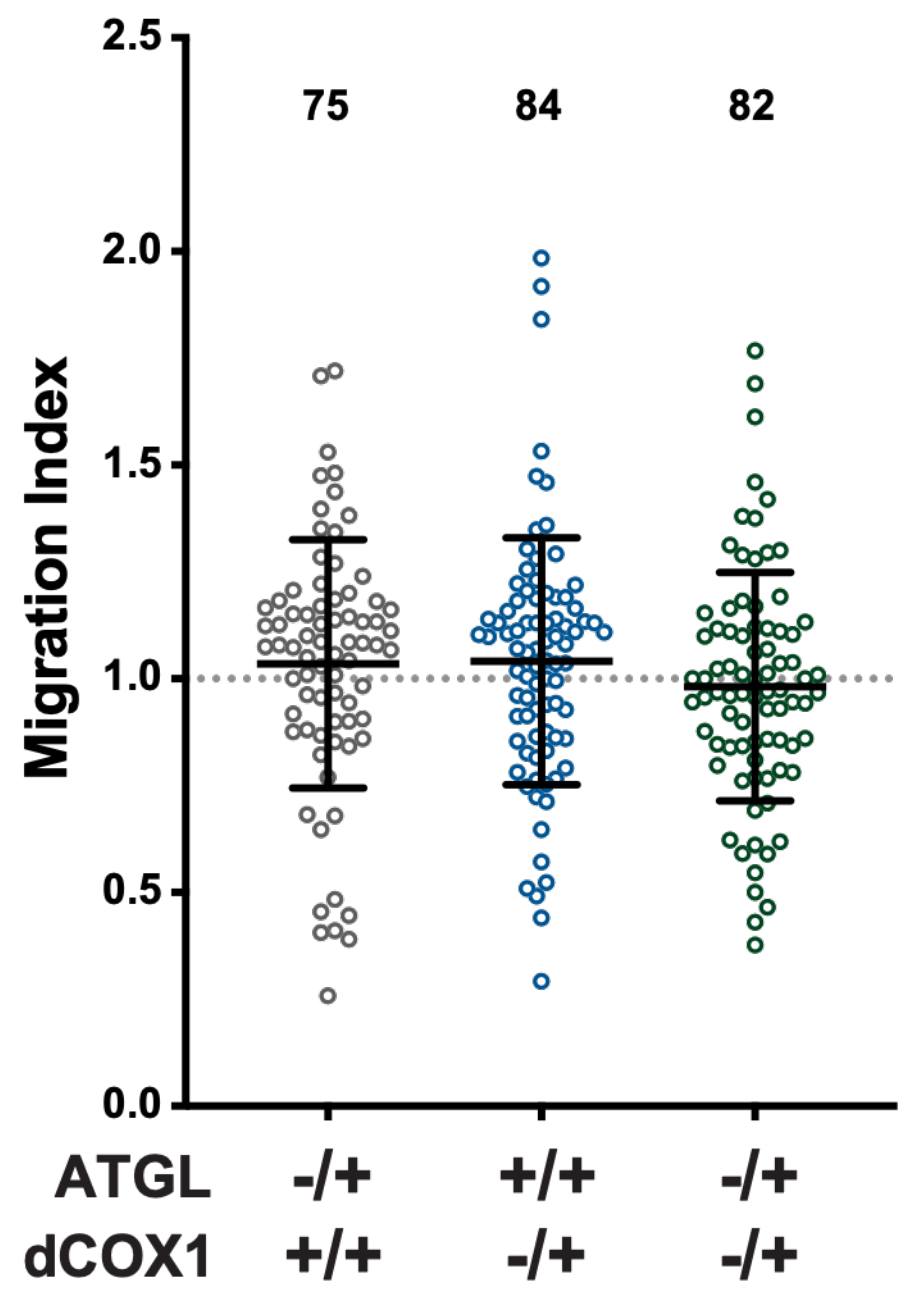

Fig. S8. ATGL and dCOX1 do not genetically interact to promote on-time border cell migration. Graph of migration index for the following genotypes: *ATGL*-/+ (*bmm*$^{1/+}$), *dCOX1*-/+ (*pxt*^{f01000}/+), *ATGL*-/+; *dCOX1*-/+ (*bmm*^{1}/+; *pxt*^{f01000}/+). Circle = individual border cell cluster, n = number of border cell clusters. The dotted line = on-time border cell migration, solid black lines = means, error bars = SD, and ns > 0.05, one-way ANOVA with Tukey multiple comparison test. Co-reduction of both ATGL and dCOX1 does not delay border cell migration, as the border cells from double heterozygotes of ATGL and dCOX1 migrate on time.

Development • Supplementary information

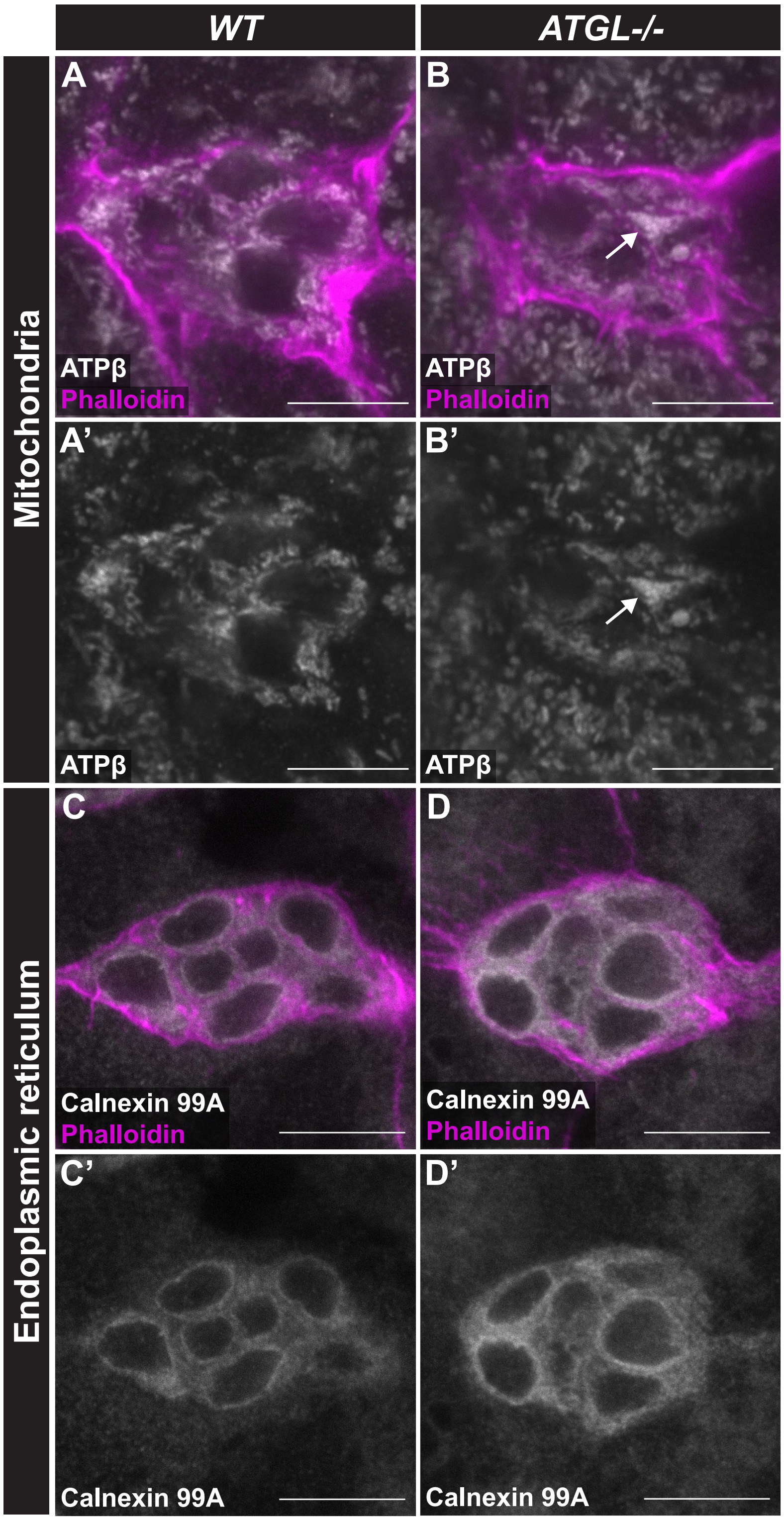

Fig. S9. Loss of ATGL disrupts mitochondrial but not ER morphology in the border cells. (A-B). Maximum projections of 3 confocal slices of S9 follicles stained for mitochondria (ATPβ)

in white and F-actin (Phalloidin) in magenta. White arrows indicate mitochondrial aggregates in the border cell cluster. Images brightened by 50% to increase clarity. Black boxes placed behind panel and channel labels to increase legibility. Scale bars = 10 µm. **(A-A')** wild-type (*yw*). **(B-B')** ATGL-/- (*bmm^1*/*bmm^1*). **(C-D)**. Maximum projections of 3 confocal slices of S9 follicles stained for endoplasmic reticulum (Calnexin 99A) in white and F-actin (Phalloidin) in magenta. Images brightened by 25% to increase clarity. Black boxes placed behind channel labels to increase legibility. Scale bars = 10 µm. **(C-C')** wild-type (*yw*). **(D-D')** ATGL-/- (*bmm^1*/*bmm^1*). Loss of ATGL results in aberrant mitochondrial clustering in the border cells **(A-B')** but does not alter the morphology of the endoplasmic reticulum **(C-D')**.

Development • Supplementary information

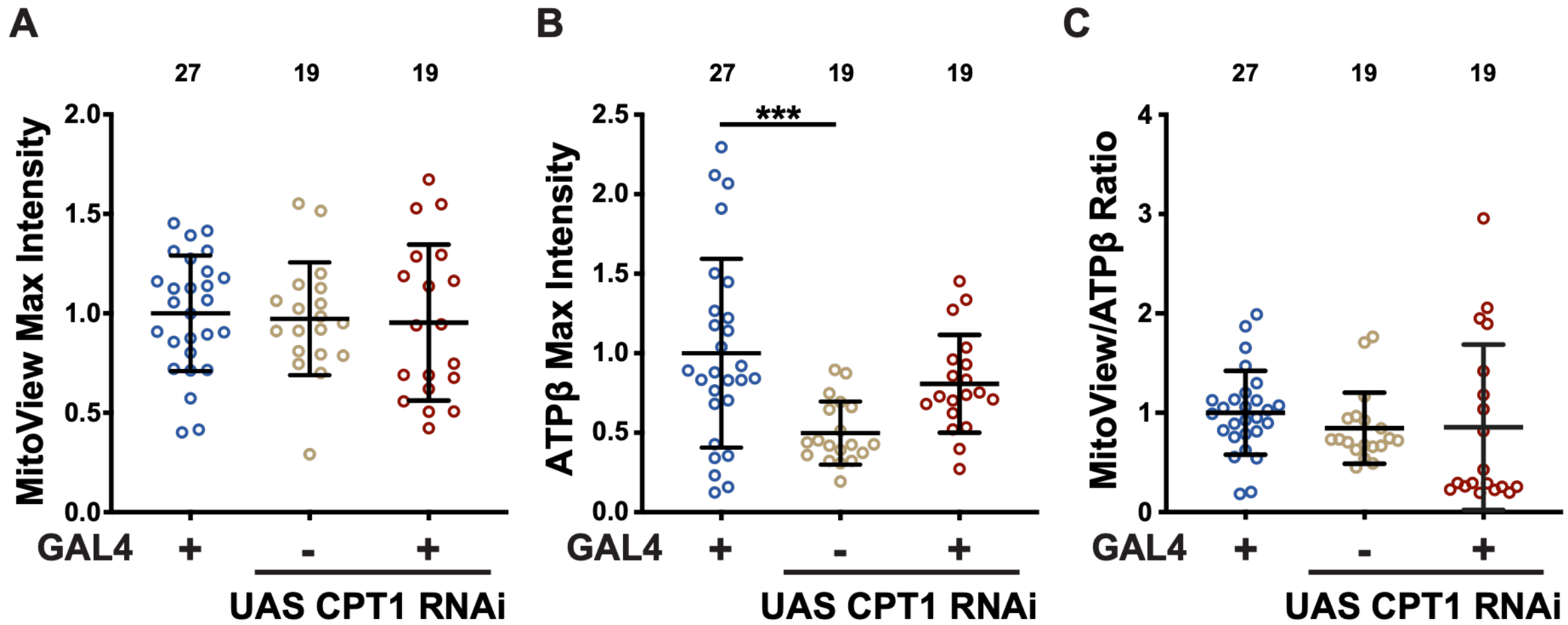

Fig. S10. Knockdown of CPT1 in the border cells does not alter mitochondrial function in the border cell cluster. (A-C). Quantification of maximum fluorescence intensity of MitoView **(A)**, maximum fluorescence intensity of ATPβ **(B)**, and ratio of MitoView to ATPβ signal **(C)** in the border cell cluster for the indicated genotypes: GAL4 control (*slbo GAL4/+*), CPT1 RNAi control (*UAS whd RNAi/+*), and border cell knockdown of CPT1 (*slbo GAL4/UAS whd RNAi*). Circle = individual border cell cluster, n = number of border cell clusters, line = mean, error bars = SD, and ns > 0.05, *** $p < 0.001$, one-way analysis of variance (ANOVA) with Tukey multiple comparison test. Border cell knockdown of CPT1 does not alter mitochondrial function **(A-C)** in the border cell cluster.

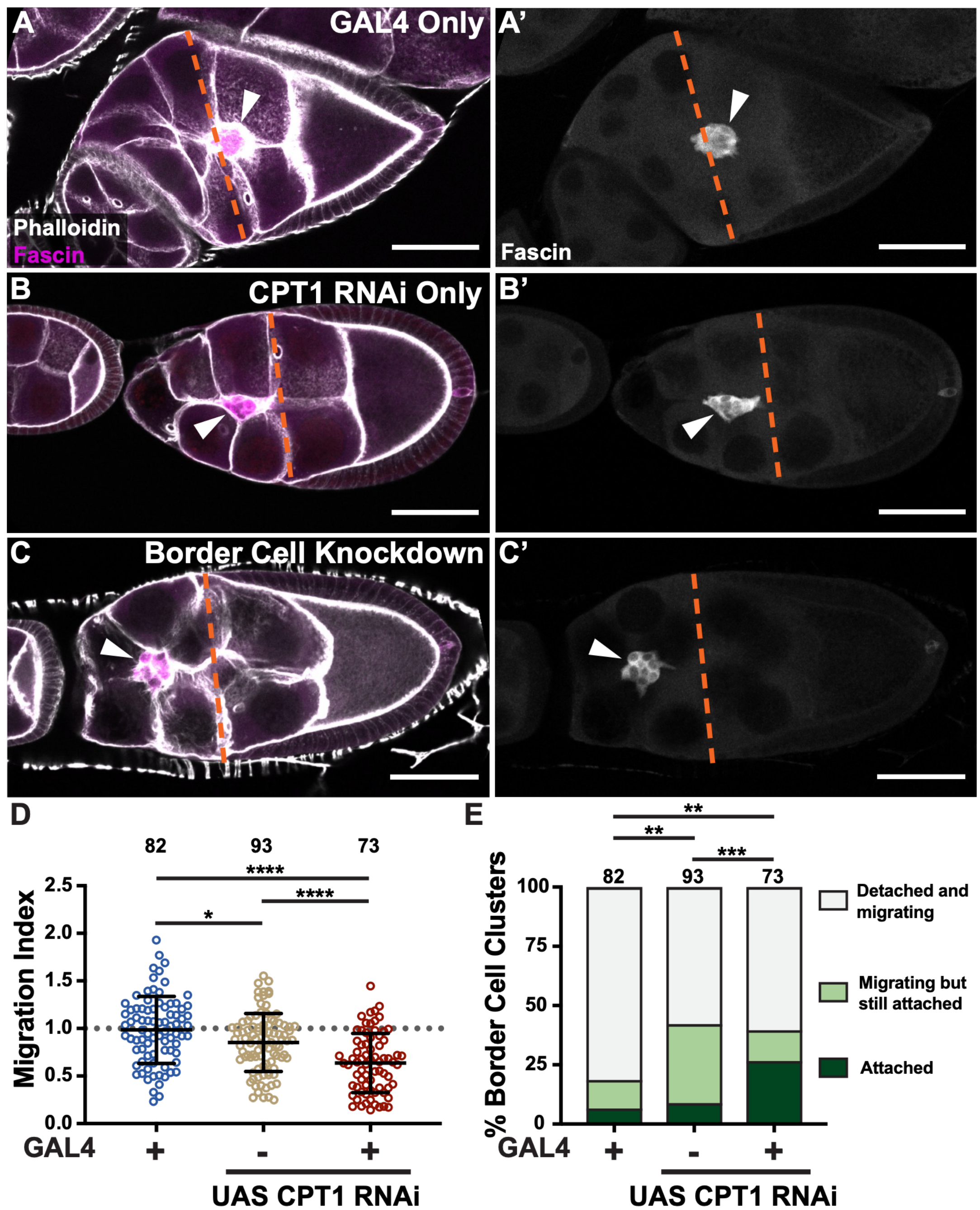

Fig. S11. CPT1 is required in the border cells for detachment and on-time migration. (A-C'). Maximum projections of 3 confocal slices of S9 follicles stained for Fascin (border cells) in magenta and F-actin (Phalloidin) in white. White arrowheads indicate the border cell cluster, and orange dashed lines indicate the anterior edge of the outer follicle cells. Images brightened by 50% to increase clarity. Black boxes placed behind panel and channel labels to increase

legibility. Scale bars = 50 µm. (A, A') GAL4 control (slbo GAL4/+). (B, B') CPT1 RNAi control (*UAS whd RNAi*/+). **(C, C')** Border cell knockdown of CPT1 (*slbo GAL4/UAS whd RNAi*). **(D).** Graph of migration index for the indicated genotypes. Circle = individual border cell cluster, n = number of border cell clusters. The dotted line = on-time border cell migration, solid black lines = means, error bars = SD, and ns > 0.05, * p < 0.05, **** p < 0.0001, one-way analysis of variance (ANOVA) with Tukey multiple comparison test. **(E).** Quantification of border cell detachment defects for the indicated genotypes. Border cell clusters categorized as detached and migrating (light grey), migrating but still attached (light green), or attached (dark green). *n* = number of border cell clusters, and ns > 0.05, ** p < 0.01, *** p < 0.001, Pearson's Chi-squared test with Monte Carlo simulation. Border cell knockdown of CPT1 delays border cell migration during S9 **(C, D)** and impairs detachment of the border cell cluster from the follicular epithelium **(E)**.

Table S1. Reagents Table. List of reagents used in the study.

Available for download at
https://journals.biologists.com/dev/article-lookup/doi/10.1242/dev.205486#supplementary-data

Table S2. Genotypes by figure. List of genotypes shown in each figure panel.

Available for download at
https://journals.biologists.com/dev/article-lookup/doi/10.1242/dev.205486#supplementary-data

Table S3. Raw data. Raw data used for all quantifications presented in both the primary and supplemental figures.

Available for download at
https://journals.biologists.com/dev/article-lookup/doi/10.1242/dev.205486#supplementary-data

Development • Supplementary information

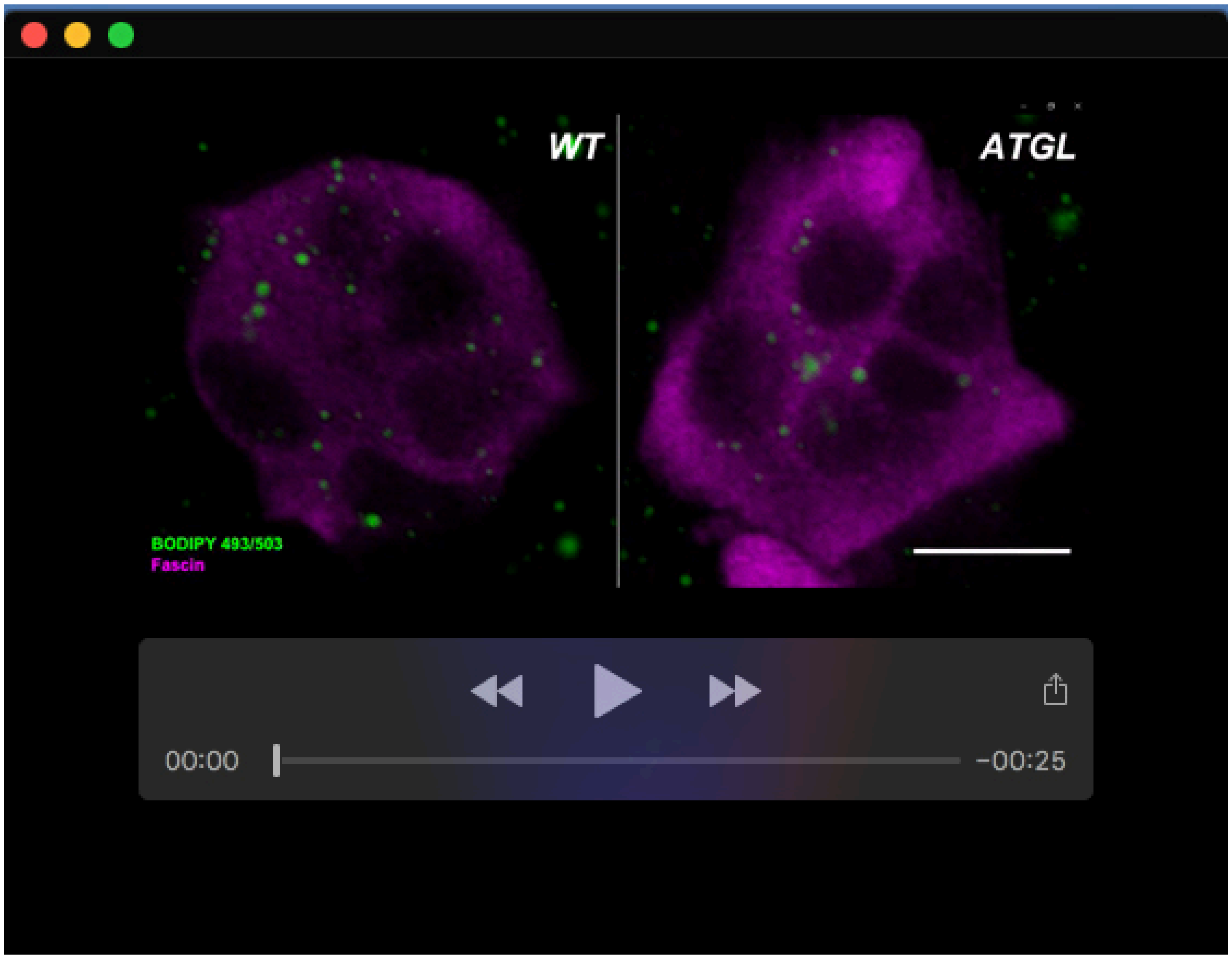

Movie 1. Loss of ATGL results in larger LDs within the BC cluster. Movie of 42 confocal slices of S9 follicles stained for LDs (BODIPY 493/503; green) and Fascin (BCs; magenta). Images brightened 30%. Scale bars = 10 µm. Left: *wild-type* (*yw*). Right: *ATGL-/-* (*bmm*^{1}/*bmm*^{1}). Loss of ATGL results in larger LDs within the BC cluster relative to wild-type.

Development • Supplementary information
