# Supplementary material for: ATGL-mediated lipid droplet lipolysis promotes collective cell migration in Drosophila
Source: Development. 2026 Aug 20;153(17):dev205486. doi: 10.1242/dev.205486 (PMC13539429; doi:10.1242/dev.205486)
Supplement: Supplementary file 2 [file develop-153-205486_review_history.pdf]

ATGL-mediated lipid droplet lipolysis promotes collective cell migration in *Drosophila*

Israel J. Wipf, Emma J. Lowden, Michelle S. Giedt, Julia R. Dorale, Kennedy F. Godfredsen and Tina L. Tootle

DOI: 10.1242/dev.205486

Editor: Irene Miguel-Aliaga

Review timeline

Original submission:	7 January 2026
Editorial decision:	4 February 2026
First revision received:	1 June 2026
Editorial decision:	17 July 2026
Second revision received:	20 July 2026
Accepted:	22 July 2026

Original submission

First decision letter

MS ID#: dev.205486

MS TITLE: ATGL-mediated lipid droplet lipolysis promotes collective migration in Drosophila

AUTHORS: Israel J. Wipf, Emma J. Lowden, Michelle S. Giedt, Julia R. Dorale, Kennedy F. Godfredsen and Tina L. Tootle

Dear Dr Tootle,

I have now received all the referees' reports on the above manuscript, and have reached a decision. The referees' comments are appended below.

As you will see, the referees express considerable interest in your work, but have some significant criticisms and recommend a substantial revision of your manuscript before we can consider publication. If you are able to revise the manuscript along the lines suggested, which may involve further experiments, I will be happy receive a revised version of the manuscript. Your revised paper will be re-reviewed by one or more of the original referees, and acceptance of your manuscript will depend on your addressing satisfactorily the reviewers' major concerns. Please also note that Development will normally permit only one round of major revision. If it would be helpful, you are welcome to contact us to discuss your revision in greater detail. Please send us a point-by-point response indicating your plans for addressing the referees' comments, and we will look over this and provide further guidance.

Please attend to all of the reviewers' comments and ensure that you upload both a 'clean' version of your Word file, along with a highlighted version clearly showing where you have made changes in the revised manuscript. Please avoid using 'Tracked changes' in Word files as these are lost in PDF conversion. I should be grateful if you would also provide a point-by-point response detailing how you have dealt with the points raised by the reviewers in the 'Response to Reviewers' box. If you do not agree with any of their criticisms or suggestions please explain clearly why this is so.

Reviewer 1

© 2026. Published by The Company of Biologists under the terms of the Creative Commons Attribution License (https://creativecommons.org/licenses/by/4.0/).

SUMMARY OF THE ADVANCE MADE IN THIS PAPER AND ITS POTENTIAL SIGNIFICANCE TO THE FIELD

Collective cell migration is essential for proper development, wound repair, and the dissemination of many cancers, and it requires the coordinated action of multiple intracellular processes. Previous studies have implicated lipid droplet (LD) homeostasis in cell migration and invasion in cancer. In this work, the authors use the migration of border cells (BCs) of the follicular epithelium in the Drosophila ovary as a model system to investigate the role of LDs in collective cell migration. The authors analyze LD dynamics during BC migration and find that both the number and volume of LDs change dynamically as migration proceeds, suggesting that lipids stored in LDs are mobilized during this process. As expected, they show that the enzyme adipose triglyceride lipase (ATGL) promotes lipid mobilization from LDs. Moreover, they demonstrate that both increased and decreased ATGL levels result in BC migration defects, including delayed migration and failure of detachment. Based on these findings, the authors propose that BC migration requires tight regulation of lipids stored in LDs. In addition, they report that loss of ATGL alters mitochondrial morphology and behavior.
Overall, this is an interesting and potentially valuable study that advances our understanding of the role of LD homeostasis in collective cell migration and suggests that this requirement may be conserved across species. However, in my view, the work requires further experimental support and analysis prior to publication. Additional experiments and in vivo analyses are needed to strengthen the mechanistic links between lipid droplet dynamics, ATGL function, mitochondrial homeostasis, and collective cell migration.

SUGGESTIONS TO AUTHORS
Major concerns
1. The authors show that LDs are present in BCs during migration. However, based on the images provided, LDs do not appear to be uniformly distributed among all BCs. Is there any positional bias in LD abundance within the migrating cluster? For example, how does LD presence compare between outer BCs and polar cells, which are non-migratory? Are LDs absent from polar cells? This is difficult to assess with the current fascin staining. Moreover, since the authors use the cell-permeable fluorescent dye BODIPY 493/503 to detect LDs, could they analyze LD dynamics using live imaging? A more detailed and dynamic characterization of LD behavior would help clarify their role during BC migration.

2. It is unclear how to interpret the increase in BC cluster size observed in ATGL mutants when cluster length is unaffected. Does this reflect an increase in individual BC size, or does it indicate reduced compaction within the cluster, leading to an apparent increase in cluster area? Clarification and quantitative analysis would be helpful.

3. Because BC migration is a highly dynamic process, migration defects should ideally be analyzed using live imaging, a widely used approach in BC migration studies. This work would greatly benefit from such analyses, as they would allow assessment of key aspects of collective migration, including detachment defects, protrusion dynamics, and cluster rotation. In addition, live imaging may reveal phenotypic differences between ATGL downregulation and ATGL overexpression, even if the final outcomes—delayed migration and detachment failure—appear similar.

4. The differences in LD phenotypes observed between ATGL null mutants (larger LDs with unchanged number) and BC-specific ATGL knockdown (increased LD number without changes in volume) require further analysis. The biological significance of these distinct phenotypes should be addressed.

5. The authors show that loss of ATGL causes changes in mitochondrial morphology and function. However, the contribution of mitochondrial defects to the role of LD homeostasis in BC migration remains unclear. Do mitochondrial perturbations similar to those observed upon ATGL manipulation independently affect BC migration? Additional experiments would be needed to establish a functional link between mitochondrial alterations and the observed migration defects.

Reviewer 2

© 2026. Published by The Company of Biologists under the terms of the Creative Commons Attribution License (https://creativecommons.org/licenses/by/4.0/).

The overall goal of this paper is to test if and how lipid droplets (LD) might contribute to border cell migration in Drosophila, an in vivo model of collective cell migration that is used to determine processes that might impact cell migration in contexts such as metastasis.
The authors first show that LD are normally present in border cells during development. They go on to show that Drosophila adipose triglyceride lipase (ATGL) homolog brummer (bmm) regulates border cell LD and that this regulation contributes to border cell migration. For these experiments they use animals with whole-body loss of ATGL and/or border cell-specific ATGL loss.
Specifically, they detected delays in on-time migration and detachment problems in migrating border cells with both gain and loss of ATGL. They also detected defects in border cell mitochondria and higher ROS due to whole-body loss of ATGL.
Together, these data show that ATGL-dependent regulation of lipolysis plays a role in supporting border cell migration, possibly via provision of fatty acids for ATP production.
Overall, the experiments are well-controlled, the data and images are presented well, and the conclusions are largely appropriate. The manuscript is also very well-written, and outlines an interesting new in vivo role for LD.
I have a few concerns that should be addressed with the data and the model as it stands, however, which I have divided into major and minor comments.

Major
(1) Cell autonomous vs non cell autonomous effects of ATGL on the phenotype. The authors present solid evidence for ATGL in regulating border cell LD, border cell migration, and mitochondrial dysfunction/ROS. The evidence that this role is cell-autonomous, however, is weaker. Indeed, border cell-specific ATGL knockdown does not cause changes in LD parameters compared to the UAS control (the ATGL KO genotype must be different from both controls). Changes to mitochondrial parameters are also only shown in ATGL mutants not in animals with border cell-specific ATGL loss. To mitigate this concern, the authors should either remove conclusions that LD changes caused by ATGL are cell-autonomous, or present stronger evidence to this effect. For example, Line 198 "knockdown of ATGL in the BCs increases LD number compared to the GAL4 control but not compared to the UAS only control" - this means there was no effect, but the authors write in Line 205 (similar text in 225) "Given that ATGL regulates LD lipolysis in a cell-autonomous manner". I recognize in the discussion the authors present a more nuanced description of these findings but this needs to be threaded throughout the entire paper so as not to give readers the impression the LD effect is cell-autonomous.
(2) Similar points for the CPT1 mutant, to ensure readers are aware this is a whole-body mutant and not showing any cell-autonomous phenotype.
(3) Normal regulation of ATGL in border cells. One key omission from the paper is the normal regulation of ATGL in border cells. This would make the case much stronger that it is border cell ATGL that might be important, rather than general defects in lipid metabolism in the whole body that happen to cause the defects observed by the authors. For example, does ATGL expression change during early, middle, and late border cell migration in line with LD changes? There are multiple bmm reporter lines and techniques that would allow the authors to assess the normal regulation of ATGL in border cells.
(4) It is unclear to me how the phenotype caused by both ATGL loss and overexpression cause exactly the same phenotype. While the authors explain this as showing that ATGL regulation needs to be accurate for proper control of lipid metabolism, it is also possible that generalized dysregulation of cellular lipid metabolism is caused in both instances and is the main reason for these broad cellular defects. This means that their model may be incomplete, as it is not simply fatty acids that are changed with ATGL loss, but also other lipids. Given that there have now been many multiomic analyses of cells and tissues lacking ATGL, which show fatty acid oxidation is one important fate of fatty acids released due to ATGL's enzymatic activity, there are a number of other potential fates for fatty acids and also lipidomic changes caused by ATGL loss. The authors should expand discussion of these studies and potential effects on border cells in the manuscript as alternative/additional mechanisms and include this possibility in their model.
(5) In the discussion the authors speculate on lipid synthesis or lipid uptake as sources for LD biogenesis. It is relatively easy to test this hypothesis via RNAi against SREBP or LpR2 to resolve this matter and would perhaps shed light into why cell-autonomous loss of ATGL does not actually change LD parameters in a cell-autonomous way.

Minor

© 2026. Published by The Company of Biologists under the terms of the Creative Commons Attribution License (https://creativecommons.org/licenses/by/4.0/).

(6) There have now been many multiomic analyses of cells and tissues lacking ATGL. While fatty acid oxidation is one important fate of fatty acids released due to ATGL's enzymatic activity, there are a number of other potential fates for fatty acids and also lipidomic changes caused by ATGL loss. The authors should expand discussion of these studies and potential effects on border cells in the manuscript as alternative/additional mechanisms.
(7) Please cite past FlyBase funding details even if funding is no longer in place.
(8) In line 27 the authors should add ATGL to the second half of the sentence "whereas BC overexpression depletes LD."
(9) Line 74 please adjust sentence to reflect LD represent a reservoir that protects the cell from excess fatty acids or lipotoxicity rather than from fatty acids.
(10) Line 82 Please change "fatty acids are broken down" to "fatty acids are metabolized".

First revision

Author response to reviewers' comments

We thank the reviewers for being enthusiastic about the study and for the suggestions for improving the manuscript. Below, we detail the revisions and provide additional clarifications (in blue) to each of the reviewer comments (in black).

Reviewer 1
1. The authors show that LDs are present in BCs during migration.

a. However, based on the images provided, LDs do not appear to be uniformly distributed among all BCs. Is there any positional bias in LD abundance within the migrating cluster? For example, how does LD presence compare between outer BCs and polar cells, which are non-migratory? Are LDs absent from polar cells? This is difficult to assess with the current fascin staining.

   We provide new data showing that the polar cells have very few LDs (Fig. S2), and that there is a polarity of LD abundance within the border cell cluster (Fig. 2). Specifically, the front (posterior) of the cluster has fewer LDs than the rear (anterior), and the lateral sides can be broken into two groups, one that is similar to the rear (lateral high) and one that is similar the front (lateral low). These findings support the idea that LD usage at the front of the cluster is higher than in the rear, and this LD depletion may contribute to leader exchange (with the replaced leader moving the lateral low side).

b. Moreover, since the authors use the cell-permeable fluorescent dye BODIPY 493/503 to detect LDs, could they analyze LD dynamics using live imaging? A more detailed and dynamic characterization of LD behavior would help clarify their role during BC migration.

   While live imaging is widely used to look at BC migration, migration is not normal ex vivo. For example, BC migration is no longer in sync with the outer follicle cell position. Additionally, we anticipate that the source of the lipids for the BC LDs is likely to be complex and include lipids coming from the fat body. Therefore, current live imaging conditions are not likely to provide an accurate reflection of LD dynamics during BC migration. We anticipate that extensive troubleshooting of ex vivo culture media will be needed to ensure LD number and size are consistent from fixed and live imaging. Therefore, these experiments, while worth doing, are beyond the scope of this paper.

2. It is unclear how to interpret the increase in BC cluster size observed in ATGL mutants when cluster length is unaffected. Does this reflect an increase in individual BC size, or does it indicate reduced compaction within the cluster, leading to an apparent increase in cluster area? Clarification and quantitative analysis would be helpful.

© 2026. Published by The Company of Biologists under the terms of the Creative Commons Attribution License (https://creativecommons.org/licenses/by/4.0/).

We provide new data showing that BC cluster height, but not length and width, is significantly increased when ATGL is lost (Fig. S3).

3. Because BC migration is a highly dynamic process, migration defects should ideally be analyzed using live imaging, a widely used approach in BC migration studies. This work would greatly benefit from such analyses, as they would allow assessment of key aspects of collective migration, including detachment defects, protrusion dynamics, and cluster rotation. In addition, live imaging may reveal phenotypic differences between ATGL downregulation and ATGL overexpression, even if the final outcomes—delayed migration and detachment failure—appear similar.

As discussed in response to Comment 1b, due to the expected challenges of optimizing lipids within the ex vivo culture conditions for live imaging, while these experiments will be important for future studies, at this time, we anticipate these experiments will likely be hard to interpret and add little additional information.

4. The differences in LD phenotypes observed between ATGL null mutants (larger LDs with unchanged number) and BC-specific ATGL knockdown (increased LD number without changes in volume) require further analysis. The biological significance of these distinct phenotypes should be addressed.

It is unclear to us what the reviewer is asking for. One explanation for the differences is that the RNAi knockdown is not sufficient or at the correct time to cause enough of a loss of ATGL function to result in the same phenotype as the null. However, we cannot assess the loss of ATGL protein in the BCs as we and others have not been able to produce an antibody to Drosophila ATGL. Further the ATGL-GFP CRISPR line can only be imaged live (antibody staining has not worked) and we can only visualize it at the surface of the follicles, preventing assessment of ATGL protein levels during BC migration. Additionally, as mentioned below in response to Reviewer 2 Major Comment 3, the ATGL-GFP line shows BC migration defects (see figure). Therefore, we feel this line may not accurately reflect ATGL protein stability and/or function, and do not include this line in the study. Without an ability to define how ATGL levels are altered during BC migration in the RNAi context, we cannot address the biological significance of the distinct phenotypes. However, we provide speculation on the potential reasons in the Discussion.

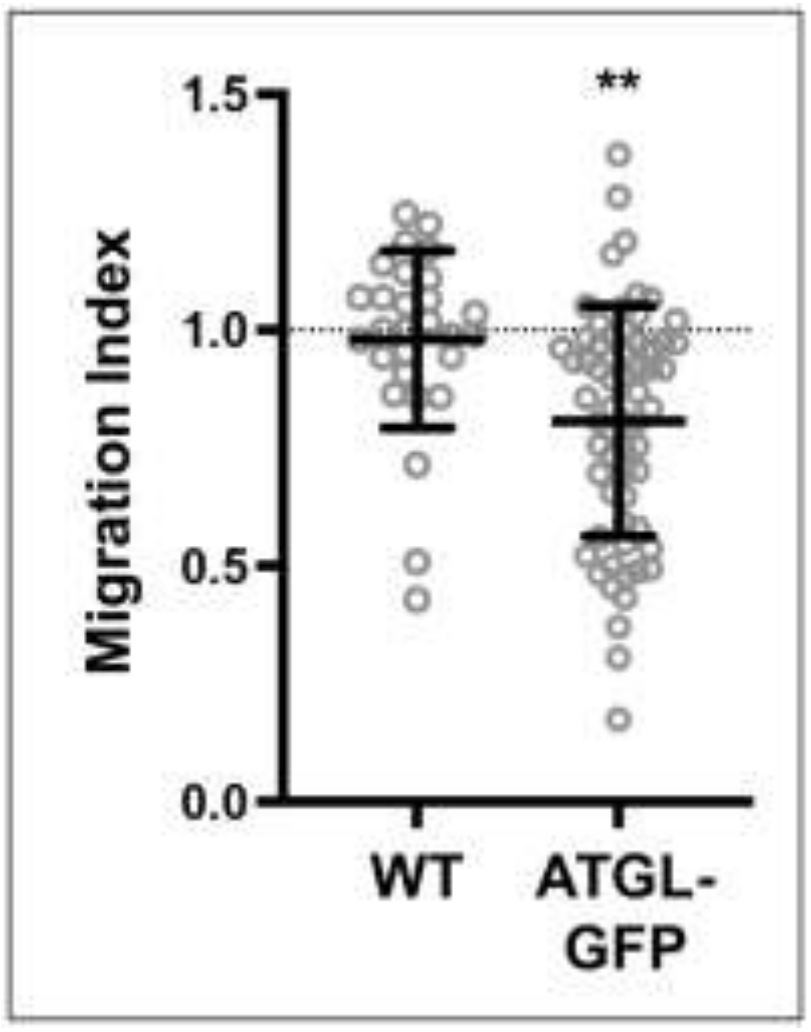

5. The authors show that loss of ATGL causes changes in mitochondrial morphology and function. However, the contribution of mitochondrial defects to the role of LD homeostasis in BC migration remains unclear. Do mitochondrial perturbations similar to those observed upon ATGL manipulation independently affect BC migration? Additional experiments would be needed to establish a functional link between mitochondrial alterations and the observed migration defects.

As mentioned in the Discussion, it has been previously shown that perturbing mitochondrial dynamics and function result in BC migration defects. However, that paper did not assess delamination. Additionally, we show that loss of CPT1, which is required for mitochondrial fatty

© 2026. Published by The Company of Biologists under the terms of the Creative Commons Attribution License (https://creativecommons.org/licenses/by/4.0/).

acid oxidation, results in a failure of the BCs to delaminate and causes changes in mitochondrial function (Figs. 8 and 9).

Reviewer 2:
Major
1. Cell autonomous vs non cell autonomous effects of ATGL on the phenotype. The authors present solid evidence for ATGL in regulating border cell LD, border cell migration, and mitochondrial dysfunction/ROS. The evidence that this role is cell-autonomous, however, is weaker. Indeed, border cell-specific ATGL knockdown does not cause changes in LD parameters compared to the UAS control (the ATGL KO genotype must be different from both controls). Changes to mitochondrial parameters are also only shown in ATGL mutants not in animals with border cell-specific ATGL loss. To mitigate this concern, the authors should either remove conclusions that LD changes caused by ATGL are cell-autonomous, or present stronger evidence to this effect. For example, Line 198 "knockdown of ATGL in the BCs increases LD number compared to the GAL4 control but not compared to the UAS only control" - this means there was no effect, but the authors write in Line 205 (similar text in 225) "Given that ATGL regulates LD lipolysis in a cell-autonomous manner". I recognize in the discussion the authors present a more nuanced description of these findings but this needs to be threaded throughout the entire paper so as not to give readers the impression the LD effect is cell-autonomous.

When we referred to the cell autonomous function of ATGL, we were referring to the known cellular function of ATGL in lipolysis within the cell it is expressed in. We did not mean to imply that our data directly shows a cell autonomous function for ATGL during BC migration. We revised the manuscript accordingly to remove this confusion.

2. Similar points for the CPT1 mutant, to ensure readers are aware this is a whole-body mutant and not showing any cell-autonomous phenotype.

We clarified the language in the manuscript to ensure the readers know the CPT1 data is for the whole-body mutant. Additionally, we assess BC RNAi knockdown of CPT1 for both migration and mitochondrial defects; however, the RNAi knockdown does not fully recapitulate the mutant phenotypes, likely due to insufficient knockdown (Fig. S10 and S11).

3. Normal regulation of ATGL in border cells. One key omission from the paper is the normal regulation of ATGL in border cells. This would make the case much stronger that it is border cell ATGL that might be important, rather than general defects in lipid metabolism in the whole body that happen to cause the defects observed by the authors. For example, does ATGL expression change during early, middle, and late border cell migration in line with LD changes? There are multiple bmm reporter lines and techniques that would allow the authors to assess the normal regulation of ATGL in border cells.

We find that the ATGL-GFP CRISPR line has a number of issues for assessing ATGL levels, localization, etc during border cell migration. First, this line cannot be assessed by antibody staining and must be assessed by live imaging, which has all the caveats described above. Additionally, by live imaging, we have only been able to observe ATGL-GFP at the surface of the follicle, meaning we can assess ATGL levels in the BCs during delamination, but cannot assess them during most of migration. Further, the ATGL-GFP line shows delayed border cell migration (see response to Reviewer 1 Comment #4), indicating it is altering ATGL stability and/or function; this raises the possibility that it also will not reflect ATGL protein dynamics. Therefore, we do not include this data in the manuscript.

4. It is unclear to me how the phenotype caused by both ATGL loss and overexpression cause exactly the same phenotype. While the authors explain this as showing that ATGL regulation needs to be accurate for proper control of lipid metabolism, it is also possible that generalized dysregulation of cellular lipid metabolism is caused in both instances and is the main reason for these broad cellular defects. This means that their model may be incomplete, as it is not simply fatty acids that are changed with ATGL loss, but also other lipids. Given that there have now been many multiomic analyses of cells and tissues lacking ATGL, which show fatty acid oxidation is one important fate of fatty acids released due to ATGL's enzymatic activity, there are a number of other potential fates for fatty acids and also lipidomic changes caused by ATGL loss. The authors

© 2026. Published by The Company of Biologists under the terms of the Creative Commons Attribution License (https://creativecommons.org/licenses/by/4.0/).

should expand discussion of these studies and potential effects on border cells in the manuscript as alternative/additional mechanisms and include this possibility in their model.

While we are unsure of which specific papers the reviewer is referring to, we added additional potential mechanisms of ATGL function to the discussion and revised the model figure accordingly. Additionally, we provide data that loss of other key LD-associated proteins does not impact border cell migration (Fig. S4), suggesting the phenotypes are specific to loss of ATGL. We also provide data that ER morphology, and thus likely function, are not impacted by altering ATGL levels (Fig. S9C-D). This finding suggests that there is not generalized dysregulation of cellular lipid metabolism as that is known to cause ER stress which significantly alters ER morphology. Additionally, loss of CPT1, the mitochondrial FA transporter, causes mitochondrial defects and failed border cell delamination that are similar to those observed when ATGL is lost (Figs. 8 and 9).

5. In the discussion the authors speculate on lipid synthesis or lipid uptake as sources for LD biogenesis. It is relatively easy to test this hypothesis via RNAi against SREBP or LpR2 to resolve this matter and would perhaps shed light into why cell-autonomous loss of ATGL does not actually change LD parameters in a cell-autonomous way.

While we agree these experiments are worth doing, we expect this to be complex (lipids coming from both de novo synthesis and uptake) and therefore is beyond the scope of the paper. Additionally, this part of the discussion has been removed in favor of discussing more potential mechanisms of ATGL function during border cell migration (as per your Major Comment #4).

Minor
6. There have now been many multiomic analyses of cells and tissues lacking ATGL. While fatty acid oxidation is one important fate of fatty acids released due to ATGL's enzymatic activity, there are a number of other potential fates for fatty acids and also lipidomic changes caused by ATGL loss. The authors should expand discussion of these studies and potential effects on border cells in the manuscript as alternative/additional mechanisms.

This comment appears to be a duplicate of Major Comment 4. Please see the above response.

7. Please cite past FlyBase funding details even if funding is no longer in place.

FlyBase funding details have been added to the Acknowledgements.

(8) In line 27 the authors should add ATGL to the second half of the sentence "whereas BC overexpression depletes LD."

The requested change was made.

(9) Line 74 please adjust sentence to reflect LD represent a reservoir that protects the cell from excess fatty acids or lipotoxicity rather than from fatty acids.

This sentence is no longer in the manuscript due to space constraints.

(10) Line 82 Please change "fatty acids are broken down" to "fatty acids are metabolized".

This sentence is no longer in the manuscript due to space constraints.

<u>Second decision letter</u>

MS ID#: dev.205486R1

MS TITLE: ATGL-mediated lipid droplet lipolysis promotes collective migration in Drosophila

© 2026. Published by The Company of Biologists under the terms of the Creative Commons Attribution License (https://creativecommons.org/licenses/by/4.0/).

AUTHORS: Israel J. Wipf, Emma J. Lowden, Michelle S. Giedt, Julia R. Dorale, Kennedy F. Godfredsen and Tina L. Tootle

Dear Dr Tootle,

I have now received all the referees reports on the above manuscript, and have reached a decision. The referees' comments are appended below.

The overall evaluation is positive and we would like to publish a revised manuscript in Development, provided that the referees' comments can be satisfactorily addressed. Please attend to all of the reviewers' comments in your revised manuscript and detail them in your point-by-point response. Whilst we are not expecting you to provide additional live imaging experiments, as previously agreed, please insert the references requested by reviewer #2.

Reviewer 1

SUMMARY OF THE ADVANCE MADE IN THIS PAPER AND ITS POTENTIAL SIGNIFICANCE TO THE FIELD

This study identifies lipid droplet homeostasis as a key regulator of collective cell migration using the Drosophila border cell model. The authors show that dynamic lipid mobilization from lipid droplets, mediated by adipose triglyceride lipase (ATGL), is required for efficient migration, as both increased and decreased ATGL activity impair border cell movement and detachment. They further link ATGL function to mitochondrial morphology, suggesting coordination between lipid metabolism and mitochondrial dynamics during migration. These findings provide new mechanistic insight into the metabolic regulation of collective cell migration and may have broader implications for understanding analogous processes in development and cancer invasion.

SUGGESTIONS TO AUTHORS

The clarifications provided by the authors and their proposed revisions substantially address most of my concerns. However, because border cell (BC) migration is a highly dynamic process, I still believe that the migration defects should be analyzed by live imaging, which is a widely used approach in BC migration studies (Major Comment 3). This would enable a more thorough assessment of key aspects of collective migration, including cluster detachment, protrusion dynamics, and cluster rotation. Moreover, live imaging may reveal phenotypic differences between ATGL downregulation and ATGL overexpression, even if both ultimately result in delayed migration and defective detachment.
The authors state that "due to the expected challenges of optimizing lipids within the ex vivo culture conditions for live imaging, while these experiments will be important for future studies, at this time, we anticipate these experiments will likely be hard to interpret and add little additional information." However, this appears to conflate two different suggestions. In Comment 1b, I proposed using live imaging to analyze lipid droplet dynamics during migration. In contrast, Major Comment 3 refers to live imaging of border cell migration itself, not of lipid droplets. There are numerous established tools for imaging BC migration in vivo, including reporters that specifically label BCs, such as tslGFP (https://doi.org/10.1371/journal.pbio.3002172), which could be readily combined with the authors' ATGL knockdown and overexpression experiments. Such experiments would provide a more comprehensive characterization of the migration phenotypes and strengthen the conclusions of the study.

Reviewer 2

SUMMARY OF THE ADVANCE MADE IN THIS PAPER AND ITS POTENTIAL SIGNIFICANCE TO THE FIELD

The authors addressed the majority of my comments, thank you.

SUGGESTIONS TO AUTHORS

The authors stated they were unsure of which studies to cite showing that ATGL affects lipids beyond TAG - here are a couple that quickly come to mind, though a deep literature dive would no

© 2026. Published by The Company of Biologists under the terms of the Creative Commons Attribution License (https://creativecommons.org/licenses/by/4.0/).

doubt identify more. Please acknowledge these studies for the published version of the manuscript. PMID: 29320510 and 34547020 and 10.1016/j.ibmb.2020.103498

Second revision

Author response to reviewers' comments

We thank the reviewers for being enthusiastic about the study, and for their suggestions for improving the revised manuscript. Both reviewers state that the revisions addressed most of their concerns. Below, we detail the revisions and provide additional clarifications (in blue) to each of the reviewer comments (in black).

Reviewer 1

The clarifications provided by the authors and their proposed revisions substantially address most of my concerns.

We are pleased that the revised manuscript has addressed most of Reviewer 1's concerns.

However, because border cell (BC) migration is a highly dynamic process, I still believe that the migration defects should be analyzed by live imaging, which is a widely used approach in BC migration studies (Major Comment 3). This would enable a more thorough assessment of key aspects of collective migration, including cluster detachment, protrusion dynamics, and cluster rotation. Moreover, live imaging may reveal phenotypic differences between ATGL downregulation and ATGL overexpression, even if both ultimately result in delayed migration and defective detachment.
The authors state that "due to the expected challenges of optimizing lipids within the ex vivo culture conditions for live imaging, while these experiments will be important for future studies, at this time, we anticipate these experiments will likely be hard to interpret and add little additional information." However, this appears to conflate two different suggestions. In Comment 1b, I proposed using live imaging to analyze lipid droplet dynamics during migration. In contrast, Major Comment 3 refers to live imaging of border cell migration itself, not of lipid droplets. There are numerous established tools for imaging BC migration in vivo, including reporters that specifically label BCs, such as tslGFP (https://doi.org/10.1371/journal.pbio.3002172), which could be readily combined with the authors' ATGL knockdown and overexpression experiments. Such experiments would provide a more comprehensive characterization of the migration phenotypes and strengthen the conclusions of the study.

While we agree that assessing the impact of altering ATGL levels on general aspects of border cell migration by live imaging (i.e. migration speed, delamination issues, protrusions) is feasible, we prefer to first determine live imaging conditions that best recapitulate the lipid droplet numbers, size, etc within the border cells; this would allow us to both assess lipid droplet dynamics and general aspects of border cell migraiton.

Multiple reasons contribute to this preference. The first reason is that ATGL's roles will depend on the lipid environment; if the lipid environment is different within standard *ex vivo* conditions, it is unclear how any observations on ATGL's functions would relate to *in vivo* border cell migration. The second reason is that border cell migration *ex vivo* is not normal; the border cells are no longer migrating in synchrony with the outer follicle cells. Perhaps once the lipid conditions are identified, *ex vivo* border cell migration will more accurately reflect *in vivo* migration. Finally, while live imaging studies would allow measurements of the impact of altering ATGL on migration speed and protrusions, such information would add more details to the story but would not change the conclusions.

Reviewer 2

© 2026. Published by The Company of Biologists under the terms of the Creative Commons Attribution License (https://creativecommons.org/licenses/by/4.0/).

The authors addressed the majority of my comments, thank you.

We are pleased that the revised manuscript has addressed most of Reviewer 2's concerns.

The authors stated they were unsure of which studies to cite showing that ATGL affects lipids beyond TAG - here are a couple that quickly come to mind, though a deep literature dive would no doubt identify more. Please acknowledge these studies for the published version of the manuscript. PMID: 29320510 and 34547020 and 10.1016/j.ibmb.2020.103498

Thank you. The previous version of the manuscript referenced PMID: 29320510 (*Salatzki et al.*, 2018). We added the other two references, in addition to the numerous currently referenced lipidomic ATGL studies, into the manuscript; these changes are highlighted in blue in the Revised Manuscript2_blue.

Third decision letter

MS ID#: dev.205486R2

MS TITLE: ATGL-mediated lipid droplet lipolysis promotes collective migration in Drosophila

AUTHORS: Israel J. Wipf, Emma J. Lowden, Michelle S. Giedt, Julia R. Dorale, Kennedy F. Godfredsen and Tina L. Tootle

Dear Dr Tootle,

I am happy to tell you that your manuscript has been accepted for publication in Development, pending our standard publication integrity checks.

© 2026. Published by The Company of Biologists under the terms of the Creative Commons Attribution License (https://creativecommons.org/licenses/by/4.0/).
